# Aberrant medial ganglionic eminence (MGE) GABAergic neurogenesis contributes to Huntington’s disease pathogenesis

**DOI:** 10.1016/j.nbd.2026.107297

**Published:** 2026-02-05

**Authors:** Aldrin E. Molero, Gnanapackiam S. Devakanmalai, Yagiz M. Altun, Teresa Jover-Mengual, Junya Zhang, Nusrat Khan, Mark F. Mehler

**Affiliations:** a The Saul R. Korey Department of Neurology, Albert Einstein College of Medicine, Bronx, NY 10461, USA; b Departamento de Fisiología, Universitat de València, Burjassot 46100, Valencia, Spain; c Department of Genetics, Albert Einstein College of Medicine, Bronx, NY 10461, USA; d Dominick P. Purpura Department of Neuroscience, Albert Einstein College of Medicine, Bronx, NY 10461, USA; e Department of Psychiatry and Behavioral Sciences, Albert Einstein College of Medicine, Bronx, NY 10461, USA; f Institute for Brain Disorders and Neural Regeneration, Albert Einstein College of Medicine, Bronx, NY 10461, USA

**Keywords:** Development, Interneurons, Fate determination, Pathogenesis, GABAergic neurons, Neurogenesis, Nkx2–1

## Abstract

Although early telencephalic interneuron dysfunction in animal models and cortical interneuron deficits in Huntington’s disease (HD) have been documented, their developmental origins and causal contributions to disease pathogenesis remain incompletely understood. Using the BACHD mouse model, we examined medial ganglionic eminence (MGE)–derived GABAergic lineage development across embryonic and early postnatal stages, integrated single-cell transcriptomic analyses of E12.5 MGE progenitors and assessed disease relevance through lineage-specific genetic rescue. At postnatal day (PND) 13, BACHD mice exhibited reduced numbers of cortical somatostatin-positive (SST^+^) and parvalbumin-positive (PV^+^) interneurons, as well as striatal PV^+^ interneurons, accompanied by a selective expansion of a Foxp2^+^ arkypallidal neuron subpopulation in the globus pallidus. By PND30, PV^+^ interneuron deficits were no longer detected, whereas cortical SST^+^ interneuron reductions persisted. Single-cell RNA sequencing revealed that mutant huntingtin disrupts early MGE neurogenic programs, with basal intermediate progenitors representing a primary site of cell vulnerability. These cells displayed coordinated repression of replication-dependent histone genes, reduced expression of the chromatin regulator *Erh*, mitochondrial and ribosomal deficits, and altered cell-cycle dynamics characterized by S-phase accumulation without increased mitotic output. Consistent with these findings, immunohistochemical analyses revealed reduced interneuron precursors within E12.5 subpallial migratory corridors and increased Nkx2–1^+^/Dlx1^+^ precursors in developing globus pallidus regions. Importantly, conditional excision of mutant *Htt* within Nkx2–1–derived MGE lineages rescued early interneuron deficits, HD-like motor impairments and striatal degeneration. Together, these findings identify disrupted MGE neurogenesis as a key developmental mechanism contributing to HD pathogenesis and highlight associated vulnerabilities as potential early-stage disease-modifying targets.

## 1. Introduction

Huntington’s disease (HD) is a progressive autosomal dominant neurodegenerative disorder caused by a CAG trinucleotide repeat expansion in exon 1 of the *Huntingtin* (*Htt*) gene. Huntingtin is a ubiquitously expressed protein essential for development and diverse cellular functions through extensive protein–protein interactions (Schulte and Littleton, 2011; Wanker et al., 2019). Although the pathogenic mutation was identified more than three decades ago, the mechanisms linking mutant huntingtin (mHTT) to disease pathogenesis remain incompletely understood. Expansion of the polyglutamine tract disrupts normal huntingtin interactions and generates aberrant ones, producing a complex interplay of loss- and gain-of-function effects that are cell-type and context dependent (Greco et al., 2022; Liu et al., 2023; Podvin et al., 2022). Despite widespread mHTT expression, HD exhibits marked differential neuronal vulnerability, with striatal spiny projection neurons showing prominent degeneration together with early functional disturbances and progressive involvement of cortical and basal ganglia circuits (Margulis and Finkbeiner, 2014; Vonsattel et al., 2011).

Although the cardinal motor, cognitive, and neurodegenerative features of HD typically emerge in midlife, converging evidence from human studies (Barnat et al., 2020; Nopoulos et al., 2011; Schultz et al., 2022; Tereshchenko et al., 2019) and animal models (Capizzi et al., 2022; Cepeda et al., 2025b; Consortium, 2017; Molero et al., 2009; Molina-Calavita et al., 2014; Zhang, 2020) indicates that mutant huntingtin also perturbs neural development. An increasing body of evidence supports a substantial contribution of early neurodevelopmental alterations to HD pathogenesis (Cepeda et al., 2025a). Consistent with this view, we previously demonstrated in the BACHD mouse model that post-developmental ablation of mutant *Htt* fails to prevent disease phenotypes, whereas developmental expression alone is sufficient to recapitulate most neurological abnormalities (Molero et al., 2016).

Normal huntingtin plays essential roles in germ layer specification, organogenesis, and neural induction (Godin et al., 2010; Nguyen et al., 2013a; Nguyen et al., 2013b). Hypomorphic expression or conditional ablation of *Htt* within subpallial regions disrupts neurogenesis of medial ganglionic eminence (MGE)-derived GABAergic lineages and leads to motor deficits and striatal degeneration later in life (Arteaga-Bracho et al., 2016; Mehler et al., 2019). In contrast, deletion of *Htt* in pallial progenitors does not cause age-dependent neuronal loss, although it reduces cortical cellularity (Dragatsis et al., 2018). Together, these findings support the idea that developmental loss-of-function mechanisms acting within subpallial GABAergic neurogenic niches, particularly the MGE, play a key role in HD pathogenesis. *Indeed, n*europathological studies in HD patients have documented interneuron abnormalities that correlate with symptom heterogeneity, disease severity, and neuronal loss (Kim et al., 2014; Mehrabi et al., 2016; Reiner et al., 2013). However, whether these alterations arise from progressive age-dependent degeneration or instead reflect disrupted developmental programs within defined subpallial progenitor populations remains unresolved. It is also unknown whether interneuron abnormalities are causally linked to later motor dysfunction and striatal degeneration.

Here, we tested the hypothesis that mutant huntingtin disrupts MGE neurogenesis and that alterations in its derived lineages causally contribute to Huntington’s disease pathogenesis. Using the BACHD mouse model, we analyzed MGE-derived GABAergic lineages across embryonic and early postnatal stages, performed single-cell transcriptomic profiling of E12.5 progenitor populations, and assessed disease relevance through lineage-specific genetic rescue. Our findings reveal impaired MGE neurogenesis and demonstrate that mutant *Htt* expression within MGE-derived lineages is necessary for the emergence of core HD phenotypes.

## 2. Methods

### 2.1. Animals

All animal procedures were conducted in accordance with the National Institutes of Health *Guide for the Care and Use of Laboratory Animals* and were approved by the Albert Einstein College of Medicine Institutional Animal Care and Use Committee (IACUC). The original BACHD mouse line (Jackson Laboratory #008197) (Gray et al., 2008) and the Dlx1-EGFP reporter line (Jackson Laboratory #030253), both originally on an FVB/N background, were backcrossed for more than ten generations onto C57BL/6 J. Hemizygous C57BL/6 J BACHD mice were subsequently bred with Dlx1-EGFP and Nkx2–1-Cre (C57BL/6 J; Jackson Laboratory #008661) lines as indicated. In previous studies, we demonstrated that mice carrying Nkx2–1-Cre efficiently and selectively drive excisional recombination across Nkx2–1–expressing forebrain and hypothalamic lineages (Mehler et al., 2019).

To validate Cre-mediated recombination in BACHD; Nkx2–1-Cre mice, this line was crossed with the Rosa26^LSL-tdTomato reporter strain (C57BL; Jackson Laboratory #007909). Forebrains from triple-transgenic embryos (BACHD; Nkx2–1-Cre; Rosa26^LSL-tdTomato) were dissociated, and tdTomato-positive and tdTomato-negative cells were isolated by fluorescence-activated cell sorting (FACS). Genomic DNA was extracted from each population and subjected to PCR analysis using two primer sets: (1) a primer pair spanning the proximal promoter through a region within the loxP-flanked exon 1 of the mHTT transgene (forward: CAGGCTAGGGCTGTCAATCA; reverse: GGCCTTCATCAGCTTTTCCAG; 329 bp), and (2) an internal positive-control confirming the BACHD genotype by targeting the human HTT exon 67 (forward: ACTGGGATGTAGAGAGGCGT; reverse: AAGATGTCAGCTGGAACGGG; 437 bp). Cells undergoing excisional recombination lack the unrecombined floxed amplicon. All mice were housed in a temperature-controlled barrier facility under a fixed 12-h light/12-h dark cycle with ad libitum access to water and food (5L0D rodent diet, PicoLab^®^). All immunohistochemical, ultrastructural, and behavioral analyses were performed using cohorts balanced for sex (1:1 male:female ratio).

### 2.2. Behavioral studies

Motor tests were administered to 12-month-old mice. Prior to testing, each mouse was acclimated to the testing room for 1 h during the light cycle. All behavioral testing and scoring were performed by experimenters blinded to genotype. *Open-field test*: Mice were placed in an opaque opalescent acrylic (254 square inches) box and allowed to freely explore the arena for 10 min, during which time voluntary locomotion (total distance traveled and speed) was recorded and thereafter analyzed using Ethovision software (Noldus). *Balance beam test*: Motor coordination deficits were measured as the number of rear limb slips made while traversing a rectangular acrylic beam. Before the test, all mice were pretrained for three consecutive days, 3-trials per day, first day on a wide plank (2.5 cm), second day on a medium-sized beam (1.5 cm) and third day on the testing beam (0.5 cm) to encourage reliable crossing. Thereafter, mice were tested for two consecutive days, 3 trials per day and the number of slips throughout the 6 trials averaged. *Rotarod test*: Each mouse was placed on the rotarod with increasing speeds, from 0 to 10 cm s^−1^ (increasing by 0.2 cm s^−1^ every 6 s) for 300 s. Each mouse received 3 trials per day for 3 consecutive days. The latency to falls off the rotarod within this period was recorded and averaged per day of testing.

### 2.3. Tissue processing and immunostaining

PND13 and PND30 mice were deeply anesthetized and transcardially perfused with ice-cold Tris-buffered saline (TBS) containing heparin (0.05 mg/mL) and sodium nitrite (0.1%; 10 mL total perfusate volume), followed by 4% paraformaldehyde (PFA; 50 mL). Brains were post-fixed in 4% PFA at 4 °C, cryoprotected in 20% sucrose at 4 °C until fully submerged and the tissue sank, embedded in cryomatrix (M-1, Epredia), flash-frozen, and stored at −80 °C. E12.5 embryos were collected by cesarean section under deep maternal anesthesia, euthanized in ice-cold TBS, and fixed in 4% PFA at 4 °C for 2.5 h. Embryonic tissue was subsequently cryoprotected in 20% sucrose, embedded, flash-frozen, and stored at −80 °C. Postnatal and embryonic specimens were cryosectioned at 30 μm and 12 μm thickness, respectively. Immunostaining was performed as previously described (Mehler et al., 2019). For cell-cycle labeling, timed-pregnant females received a single intraperitoneal (IP) injection of BrdU (200 mg/kg) 1 h prior to embryo collection. For BrdU immunodetection, heat-mediated antigen retrieval was performed in citrate buffer containing 0.05% Tween-20 for 30 min at 100 °C, followed by DNA denaturation in 2 N HCl at 37 °C for 40 min and neutralization in 0.1 M sodium borate buffer (pH 8.0) for 20 min. Primary antibodies included: ChAT (goat anti-ChAT, 1:1000, Sigma-Aldrich, #ab144), BrdU (mouse anti-BrdU, 1:500, R&D systems, #MAB7225), Foxp2 (sheep anti-Foxp2, 1:200, R&D Systems, #AF5647), GFP (chicken anti-GFP, 1:1000, Abcam, #ab13970), Lhx6 (mouse anti-Lhx6, 1:200, Santa Cruz Biotechnology, #sc-271,433), Mafb (rabbit anti-Mafb, 1:200, Cell Signaling Technology, #30919), Nkx2–1 (mouse anti-Nkx2–1, 1:100, Sigma-Aldrich, #MAB5460), NPY (rabbit anti-NPY, 1:500, Cell Signaling Technology, #D745A), phospho-Histone H3 (Rabbit anti-pHH3, 1:300, Cell Signaling Technology, #9701), PV (mouse anti-PV, 1:300, Sigma-Aldrich, #P3086), Satb1 (mouse anti-Satb1, 1:200, Santa Cruz Biotechnology, #sc-376,096), Sox3 (Goat anti-Sox3, 1:500, R&D Systems, #AF2569), SST (rabbit anti-SST, 1:1000, BMA Biomedicals, #T-4103), and VIP (mouse anti-VIP, 1:200, R&D Systems, #576721). Alexa Fluor–conjugated secondary antibodies (Invitrogen) were used at 1:1500. Images were acquired using the Apotome 3 structured illumination system in a widefield fluorescence Zeiss Axioscope microscope with an Axiocam 705 camera. All immunostaining, imaging, and quantification were performed with experimenters blinded to genotype, and sections from different genotypes were processed in parallel to minimize batch effects.

### 2.4. Electron microscopy

For electron microscopy, 12-month-old mice were perfused as described above, except that fixation was performed using 2% PFA and 2.5% glutaraldehyde in 0.1 M cacodylate buffer (pH 7.4; 50 mL). Brains were sliced into 2-mm coronal sections and post-fixed for an additional 1 h in the same solution. After rinsing in 0.1 M cacodylate buffer, samples were treated with 1% osmium tetroxide for 1 h, rinsed in buffer and distilled water, and stained with 2% uranyl acetate for 1 h. Specimens were dehydrated through graded ethanol, embedded in Epon LX112, and sectioned into ultrathin sections for imaging. Transmission electron micrographs were acquired on a JEOL 1200EX equipped with a Gatan Orius 2 K × 2 K camera. Images were collected at 3000× magnification for neuronal degeneration analyses and 8000× magnification for axonal myelination assessments.

### 2.5. Image analysis

Cortical interneurons were quantified in four serial coronal sections from the somatosensory cortex, separated by 180 μm (PND13) or 300 μm (PND30). The most anterior section corresponded to the level at which the commissural fibers of the corpus callosum begin to cross rostrally between hemispheres. The cortical region of interest (ROI) consisted of a 389-μm-wide column extending from the pial surface to the white matter boundary of the corpus callosum. Striatal interneurons were quantified within four rectangular ROIs (0.182 mm^2^ each) sampling dorsal, ventral, medial, and lateral regions of the lateral striatum across three consecutive coronal sections, each separated by 300 μm. For the globus pallidus, interneurons were quantified in two coronal sections separated by 120 μm, corresponding to bregma −0.34 and −0.46 mm. The globus pallidus ROI encompassed the entire nucleus, delineated by dense Dlx1-EGFP reporter expression. Quantifications in E12.5 embryos included the following regions: (1) lateral ganglionic eminence (LGE): three circular ROIs (7482 μm^2^ each) within the mantle, spaced 60 μm apart; (2) ventral pallium (VP): three ROIs (40,426–96,322 μm^2^) encompassing the entire VP, delineated by Dlx1-EGFP expression, spaced 60 μm apart; (3) globus pallidus primordium, two circular ROIs (33,528 μm^2^ each) within the caudal aspect of the medial ganglionic eminence mantle, spaced 60 μm apart; (4) Nkx2–1^+^ MGE germinative zone, three ROIs, consisting of 50-μm-wide columns encompassing the entire ventricular and subventricular zones, separated by 60 μm and identified by Nkx2–1 and Dlx1-EGFP expression; and (5) BrdU^+^ and pHH3^+^ cells within MGE germinative zones, quantified in four ROIs (two ventral and two dorsal), also corresponding to 38-μm-wide column extending from the apical surface to the intermediate zone (germinative region juxtaposed to subventricular zone). All cell counts were normalized to ROI area and averaged across sections for each region. For all analyses, the animal was treated as the unit of biological replication, with section- and ROI-level measurements averaged per animal prior to statistical analysis. Image acquisition and quantification were performed by experimenters blinded to genotype. For quantification of striatal neurodegeneration by transmission electron microscopy (TEM), neurons were counted in 50 randomly selected square fields (6400 μm^2^ each) per specimen. Degenerating neurons were identified based on electron-dense nuclei and cytoplasm, chromatin clumping, swelling of endoplasmic reticulum and Golgi cisternae, and plasma membrane ruffling. Myelin thickness was assessed using g-ratios, calculated from cross-sectional areas as the ratio of axon area to the total fiber area (axon plus myelin). At least 20 TEM micrographs were analyzed from deep layer VI of the somatosensory cortex in 12-month-old mice. Within each image, 7–9 axons were randomly selected using a superimposed square grid.

### 2.6. Single-cell RNA sequencing library generation and analyses

By mating two hemizygous mice, we generated a biallelic BACHD line. Genotype was first determined by qRT-PCR and subsequently confirmed by verifying the male’s ability to produce exclusively BACHD^+^ offspring when bred with wild-type (WT) females. This BACHD male, which also carried the *Dlx1-EGFP* reporter, was then crossed with WT females. Control animals were generated by breeding WT males carrying the *Dlx1-EGFP* reporter with WT females. At E12.5, BACHD^+^ embryos carrying the *Dlx1-EGFP* reporter were identified under a fluorescent lamp. Eight embryos per experimental group were harvested by cesarean section. Pairs of MGEs from each embryo were microdissected and pooled into two groups, each containing four pairs of MGEs. Accordingly, after dissociation, the sample consisted of two independent pools of cell suspensions per genotype (BACHD and WT). Each pool was fluorescence-activated cell sorted (FACS) using a MoFlo XDP high-speed cell sorter (Beckman Coulter) equipped with a 120-μm nozzle and operated at a sheath pressure of 16.6 PSI, yielding >80% cell viability. Each independent pool of single-cell suspensions (up to 10,000 cells per sample) was then separately processed for scRNA-seq library preparation using the Chromium Next GEM Single Cell 3′ v3.1 kit (10× Genomics, Pleasanton, CA), following the manufacturer’s protocol. Following cDNA amplification and library preparation, libraries were sequenced on an Illumina NextSeq 2000 with a 100-cycle P2 reagent kit. Sequencing was performed with paired-end dual-indexing parameters: 28 cycles for Read 1 (cell barcode and UMI), 10 cycles for i7 index, 10 cycles for i5 index, and 90 cycles for Read 2 (cDNA insert). On average, a depth of 26,021 reads per cell (range 20,995 – 33,813) was achieved. The estimated number of cells per sample was 11,136 (285,338,183 reads) and 11,266 (266,454,416 reads) for BACHD pools 1 and 2, respectively, and 7985 (269,995,383 reads) and 13,120 (275,456,042 reads) for WT control pools 1 and 2, respectively (Supplementary Data 1).

Analyses were performed in R (v4.4.3) primarily using the Seurat package (v5.2.0) (Hao et al., 2024; Ihaka and Gentleman, 1996). Initial processing of raw 10× Genomics count matrices began with the removal of ambient RNA contamination using the SoupX package (Young and Behjati, 2020). For each sample, the contaminant fraction was automatically estimated, and the raw count matrix was adjusted accordingly. Following ambient RNA correction, each sample was individually processed for quality control and doublet removal. The corrected counts were used to create a Seurat object, and initial filtering was applied to retain cells with at least 200 detected genes (nFeature_RNA > 200) and 500 Unique Molecular Identifiers (UMIs) (nCount_RNA > 500). Genes expressed in fewer than 10 cells were excluded from further analysis. Cells with a mitochondrial gene content exceeding 15% were discarded. Heterotypic doublets were identified and removed using the DoubletFinder algorithm (McGinnis et al., 2019). The filtered, high-quality Seurat objects from each sample were merged into a single dataset. The combined data was normalized using the NormalizeData function, and the top 2000 most variable features were identified. To mitigate the effects of unwanted technical and biological variation, the data was scaled while regressing out the number of features (nFeature_RNA), mitochondrial and ribosomal percentages, and cell cycle scores. Cell cycle scores were regressed out for clustering and integration but were retained for post hoc cell cycle–resolved analyses. To correct for batch effects between individual samples, the Harmony integration algorithm was applied to the principal component analysis (PCA) embeddings. PCA was then performed on the scaled data of the integrated object. The optimal number of Harmony-corrected principal components for downstream analysis was determined by identifying the elbow in the PCA plot.

Selected principal components were used to construct a shared nearest neighbor (SNN) graph using the FindNeighbors function in Seurat. To account for the high degree of cellular heterogeneity inherent to embryonic tissues, cluster quality was evaluated using the Ratio of Global Unshifted Entropy (ROGUE) statistic to assess cluster purity and inform refinement of unsupervised clustering (Liu et al., 2020). Cell clustering was performed using the Leiden algorithm at a resolution of 0.5. Initial clusters were further evaluated and refined using the Single-Cell Clustering Assessment Framework (SCCAF) to optimize cluster stability and classification accuracy (Miao et al., 2020). This approach resolved 29 transcriptionally distinct clusters, each representing a coherent MGE-derived cellular population suitable for downstream lineage annotation and analyses (Supplementary Data 2). To confirm proper sample integration after Harmony, we provide Supplementary Fig. S1, which displays cells colored by both sample of origin and genotype.

Clusters were visualized using Uniform Manifold Approximation and Projection (UMAP), and cell-type annotation was performed by manual curation guided by established canonical marker gene expression. Based on shared transcriptional features and lineage relationships, the 29 initial clusters were subsequently consolidated into 11 broader cell-type categories. Differential gene expression between BACHD and control embryos was assessed using pseudobulk analysis followed by DESeq2 (Supplementary Data 3), from which MA plots were generated. Gene Ontology enrichment analyses for Biological Process and Cellular Component categories were performed using ShinyGO v0.77 (Ge et al., 2020). UMAP visualizations were produced using DimPlot, gene expression patterns were displayed using FeaturePlot and VlnPlot, and expression heatmaps were generated using the pheatmap package.

Cell-cycle state was inferred using Seurat Cell CycleScoring, generating continuous S.Score and G2M.Score values and assigning cells to G1, S, or G2/M phases by standard classifier criteria. Analyses were restricted to defined progenitor clusters, with phase proportions compared by genotype. Phase-restricted pseudobulk profiles were generated by aggregating cells within each phase and embryo, followed by differential-expression analysis (Supplementary Data 4). Coordinated histone expression was quantified using AddModuleScore based on histone genes empirically identified as downregulated in BACHD basal progenitors by pseudobulk analysis.

Single-cell histone module scores were calculated using AddModuleScore. A “downregulated histone module” was defined empirically as the set of histone genes identified as significantly repressed in BACHD basal progenitors by genotype-level pseudobulk analysis, independent of cell-cycle stratification. In parallel, a second module restricted to Histone-1 family members was analyzed to assess linker histone regulation. Module scores were compared between genotypes within inferred cell-cycle phases for post hoc, cell cycle–resolved analyses.

Operationally, in this study, the term neural progenitors refers to developing cells that are actively cycling or have only recently exited the cell cycle and are characterized by high expression of canonical progenitor markers, including *Ascl1*, *Fabp7*, *Gadd45g*, and *Dll3*. In contrast, neural precursors denote postmitotic developing cells in which progenitor-marker expression is no longer dominant and that are migrating toward their differentiation niches. We additionally define a post-mitotic progenitor state to denote newly cell cycle–exited cells that retain expression of progenitor-associated transcription factors (e.g., *Sox2*, *Ascl1*), while initiating early GABAergic-lineage programs (e.g., *Sox6*, *Lhx6*) and downregulating cell cycle transcripts, representing a short-lived transitional stage prior to full precursor maturation.

### 2.7. Statistical analysis

For comparisons of means, minimal sample sizes required to achieve adequate statistical power were estimated using G*Power software (Ferrante, 2009). Effect sizes and variances were calculated post hoc from data reported in previous studies with similar experimental designs (Arteaga-Bracho et al., 2016; Mehler et al., 2019; Molero et al., 2016; Molero et al., 2009). For all analyses, the animal was treated as the unit of biological replication. When applicable, measurements obtained from multiple sections, regions of interest, or cells were averaged per animal prior to statistical testing. Mean comparisons between independent experimental groups were performed using unpaired two-tailed Student’s *t*-tests or one-way ANOVA for data fitting a Gaussian distribution, and Mann–Whitney *U* or Kruskal–Wallis tests for non-Gaussian distributions. Normality was assessed using the Kolmogorov–Smirnov test. Latency-to-fall measurements in the rotarod test across three days were analyzed with two-way repeated-measures ANOVA. Post hoc multiple comparisons were performed using Tukey’s or Dunnett’s tests with adjustment for multiple comparisons. Proportional data (e.g., percentage of cells per cluster, proportion of degenerating cells) were analyzed using Fisher’s exact test. For axonal myelination, the relationship between axonal area and g-ratio was examined by scatterplot and compared across groups using linear regression analyses. Statistical analyses were performed using GraphPad Prism version 10.6.0 for Windows.

Post hoc power analyses were conducted using G*Power. For one-way ANOVA comparisons (α = 0.05), analyses based on observed effect sizes indicated achieved power > 0.99 for Open Field distance traveled (*F* = 5.03; *n* = 47; three groups), Open Field speed (*F* = 4.93; *n* = 47; three groups), and Balance Beam performance (*F* = 6.29; *n* = 31; three groups). Similarly, post hoc power analysis for rotarod performance using a repeated-measures ANOVA (α = 0.05), based on the observed effect size (*F* = 5.34; *n* = 32; three groups; three repeated measurements; assumed within-subject correlation = 0.5), indicated achieved power > 0.99. Together, these analyses confirm that the study was sufficiently powered to detect the observed effects.

## 3. Results

### 3.1. BACHD mice exhibit reductions in several classes of MGE-derived interneurons in both cortex and striatum at PND13

**Fig. 1.**
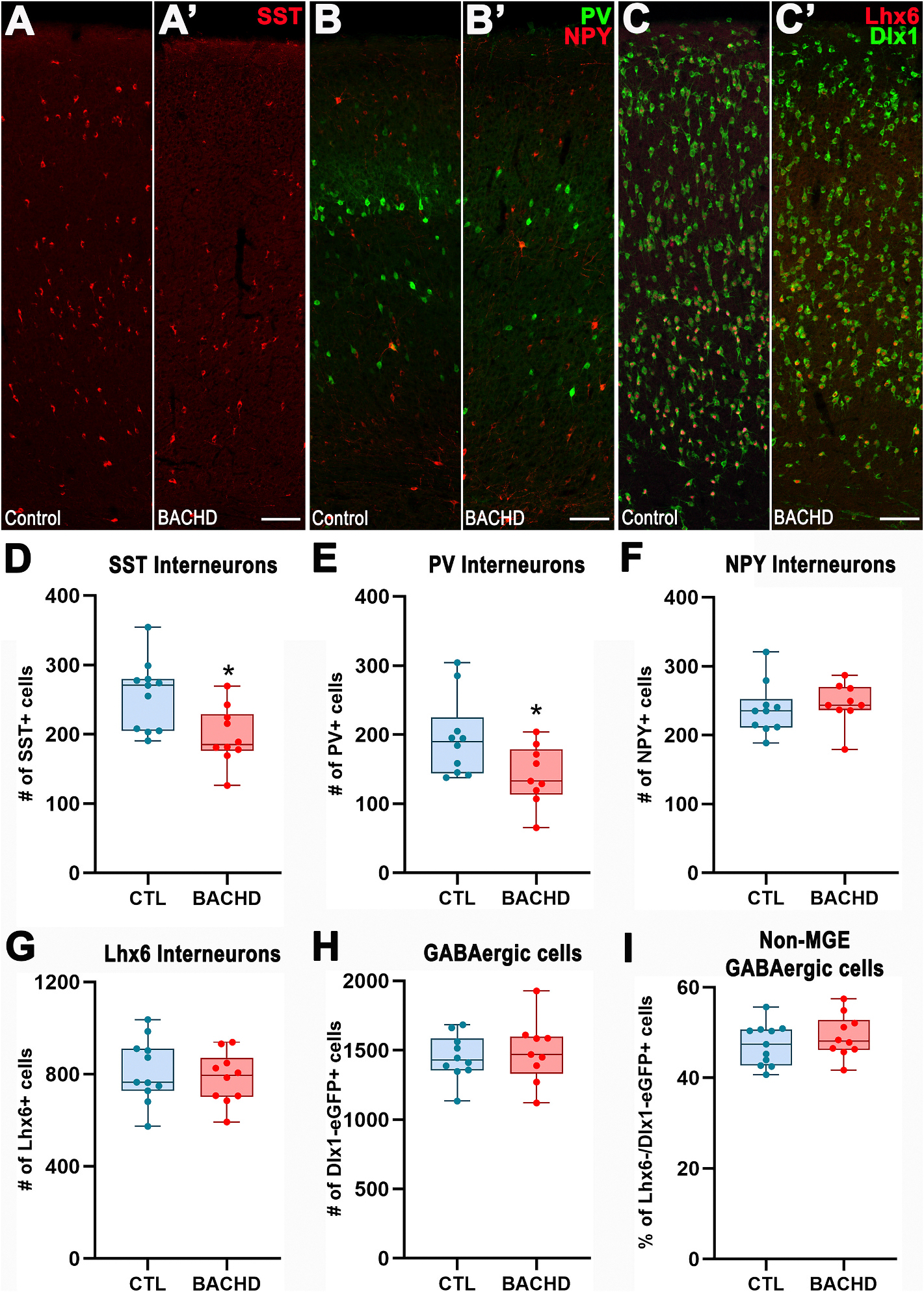
SST- and PV-expressing interneurons, but not Lhx6^+^ cells, are reduced in the PND13 BACHD cortex. (A–C) Representative immunofluorescence images of the somatosensory cortex from PND13 mice stained for SST (A), PV and NPY (B), and Lhx6 together with EGFP driven by the *Dlx1* promoter (Dlx1; C). (D-I) Quantification of SST^+^ (D), PV^+^ (E), NPY^+^ (F), Lhx6^+^ (G), EGFP driven by the *Dlx1* promoter (H; surrogate marker for GABAergic neurons), and the percentage of Dlx1^+^ cells not expressing Lhx6 (I), representing the proportion of non-medial ganglionic eminence (MGE)-derived cortical GABAergic neurons. Data are presented as box-and-whisker plots showing median, interquartile range, and minimum/maximum values. Individual data points represent biological replicates. Statistical analysis was performed using unpaired Student’s *t*-test; *p* < 0.05 was considered significant. Scale bars: 100 μm (A–C).

Interneurons play critical roles in circuit plasticity by modulating the activity and connectivity of developing neural circuits during the early postnatal period (Ferrer and De Marco Garcia, 2022; Reh et al., 2020). To assess interneuron development during this critical maturational window, we analyzed the cortex and striatum of PND13 mice, a time point when a large proportion of maturing medial ganglionic eminence (MGE)-derived interneurons express their characteristic neurochemical subtype markers. In the cortex, the two major MGE-derived interneuron subtypes, somatostatin-positive (SST^+^) and parvalbumin-positive (PV^+^) cells were significantly reduced in BACHD mice compared to controls (SST^+^: Control: 256.1 ± 15.1, *n* = 11; BACHD: 197.7 ± 12.9, *n* = 10; *t*_19_ = 2.91, *p* = 0.009; Fig. 1A/A’,D; PV^+^: Control: 195.2 ± 18.3, *n* = 10; BACHD: 141.5 ± 14.4, *n* = 9; *t*_17_ = 2.27, *p* = 0.036; Fig. 1B/B′,E). Deficits in interneuron number were present in both infragranular (SST^+^: Control: 183 ± 41.9, *n* = 11; BACHD: 144.6 ± 33.2, *n* = 10; *t*_19_ = 2.31, *p* = 0.032 Supplementary Fig. S2A; PV^+^: Control: 104.9 ± 27.5, *n* = 10; BACHD: 79.7 ± 22.4, *n* = 9; *t*_17_ = 2.17, *p* = 0.044; Supplementary Fig. S2C) and supragranular (SST^+^: Control: 73.11 ± 11.28, *n* = 11; BACHD: 50.1 ± 14.1, *n* = 10; *t*_19_ = 3.61, *p* = 0.002; Supplementary Fig. S2B; PV^+^: Control: 90.3 ± 33.4, *n* = 10; BACHD: 61.8 ± 22.7, *n* = 9; *t*_17_ = 2.15, *p* = 0.046; Supplementary Fig. S2D) cortical layers. By contrast, the number of cortical neuropeptide Y-positive (NPY^+^) interneurons, a subtype only partially derived from the MGE, did not differ significantly between groups (Control: 237.8 ± 12.1, *n* = 10; BACHD: 246.0 ± 10.2, *n* = 9; *t*_19_ = 0.51, *p* = 0.618; Fig. 1B/B′,F). Despite the reductions in SST^+^ and PV^+^ interneurons, the total number of cortical GABAergic cells expressing the pan-MGE marker, Lhx6 was comparable between BACHD and control mice (Control: 815.6 ± 41.7, *n* = 11; BACHD: 782.8 ± 35.0, *n* = 10; *t*_19_ = 0.59, *p* = 0.559; Fig. 1C/C′,G). Similarly, the number of cells expressing the pan-GABAergic reporter, Dlx1-EGFP did not differ significantly between groups (Control: 1450 ± 51.6, *n* = 10; BACHD: 1490 ± 76.5, *n* = 9; *t*_17_ = 0.44, *p* = 0.668; Fig. 1C/C′,H). Overall, these data highlight the presence of significant deficits in SST^+^ and PV^+^ cortical interneurons in BACHD mice. However, the comparable numbers of cells expressing pan-MGE and pan-GABAergic markers indicate that a substantial fraction of these cells remain devoid of distinct mature neurochemical marker expression.

To estimate the proportion of PND13 non-MGE-derived cortical GABAergic cells, we calculated the number of Dlx1-EGFP^+^/Lhx6^−^ cells. This fraction was also similar between genotypes (Control: 47.3 ± 1.4, *n* = 11; BACHD: 49.2 ± 1.5, *n* = 10; *t*_19_ = 0.93, *p* = 0.362; Fig. 1I). Consistently, the numbers of vasoactive intestinal peptide-positive (VIP^+^) and calretinin-positive (CR^+^) interneurons, two non-MGE-derived subtypes, were also unchanged (VIP^+^: Control: 10.5 ± 2.7, *n* = 7; BACHD: 12.9 ± 2.3, *n* = 7; Mann–Whitney *U* = 19, *p* = 0.535; CR^+^: Control: 3.3 ± 1.07, *n* = 7; BACHD: 4.21 ± 0.98, *n* = 7; Mann–Whitney *U* = 16.5, *p* = 0.33).

**Fig. 2.**
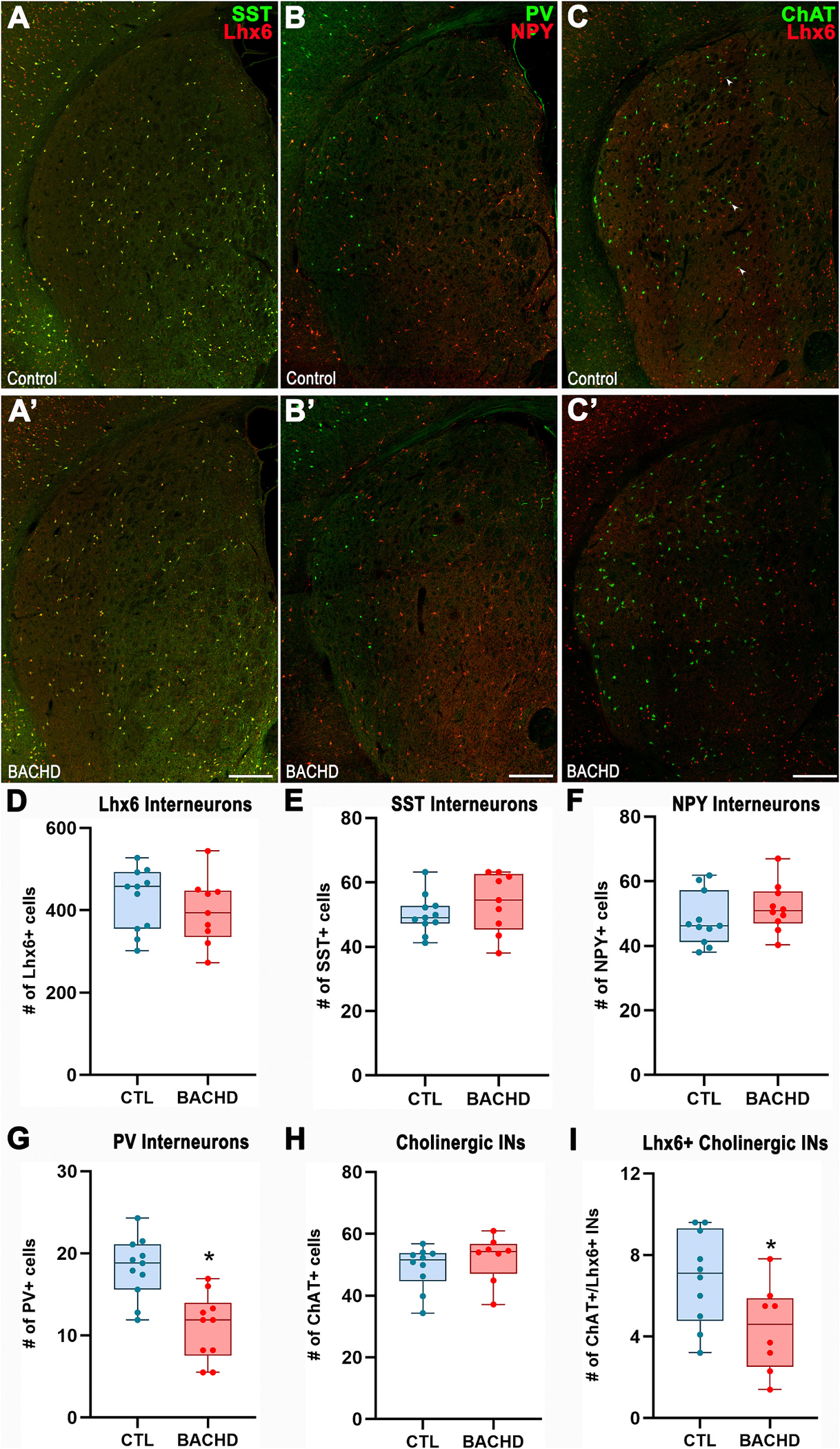
PV- and Lhx6/ChAT-expressing interneurons are reduced in the PND13 BACHD striatum. (A–C′) Representative immunofluorescence images of the striatum from PND13 mice stained for SST and Lhx6 (A/A′), PV and NPY (B/B′), and ChAT and Lhx6 (C/C′). (D-I) Quantification of Lhx6^+^ (D), SST^+^ (E), NPY^+^ (F), PV^+^ (G), ChAT^+^ (H), and Lhx6^+^/ChAT^+^ (I) interneurons. Data are shown as box-and-whisker plots indicating the median, interquartile range, and minimum/maximum values. Individual data points represent biological replicates. Statistical analysis was performed using unpaired Student’s *t*-test; *p* < 0.05 was considered statistically significant. Scale bars: 300 μm (A–C).

We next examined MGE-derived interneurons in the PND13 striatum (Fig. 2). The overall number of Lhx6^+^ cells, as well as SST^+^ and NPY^+^ interneurons, were comparable between control and BACHD mice (Lhx6^+^: Control: 426.4 ± 22.86, *n* = 11; BACHD: 397.9 ± 21.2, *n* = 9; *t*_18_ = 0.81, *p* = 0.428; Fig. 2A,A’,D; SST^+^: Control: 50.1 ± 6.1, *n* = 11; BACHD: 53.7 ± 9.3, *n* = 9; *t*_18_ = 1.06, *p* = 0.303; Fig. 2A,A’,E; NPY^+^: Control: 48.2 ± 2.5, *n* = 11; BACHD: 51.8 ± 2.36, *n* = 10; *t*_19_ = 1.05, *p* = 0.308; Fig. 2B,B’,F). By contrast, PV^+^ interneurons were significantly reduced in the striatum of BACHD mice compared to controls (Control: 18.2 ± 1.1, *n* = 11; BACHD: 11.02 ± 1.3, *n* = 10; *t*_19_ = 4.26, *p* = 0.0004; Fig. 2B/B′,G). The MGE also gives rise to striatal cholinergic interneurons. While the total number of cholinergic cells appeared unchanged in BACHD mice (Control: 49.1 ± 2.2, *n* = 10; BACHD: 52.1 ± 2.7, *n* = 8; *t*_16_ = 0.89, *p* = 0.387; Fig. 2C,C’,H), the number of cholinergic interneurons co-expressing Lhx6^+^ was significantly reduced in BACHD mice compared to controls (Control: 6.9 ± 0.72, *n* = 10; BACHD: 4.42 ± 0.75, *n* = 8; *t*_16_ = 2.33, *p* = 0.036; Fig. 2I). Together, these findings reveal selective striatal interneuron deficits in BACHD mice, including reduced PV^+^ interneurons and a diminished subpopulation of Lhx6^+^ cholinergic interneurons.

### 3.2. Selective expansion of Dlx1-EGFP^+^ Arkypallidal neurons in the globus pallidus of BACHD mice

**Fig. 3.**
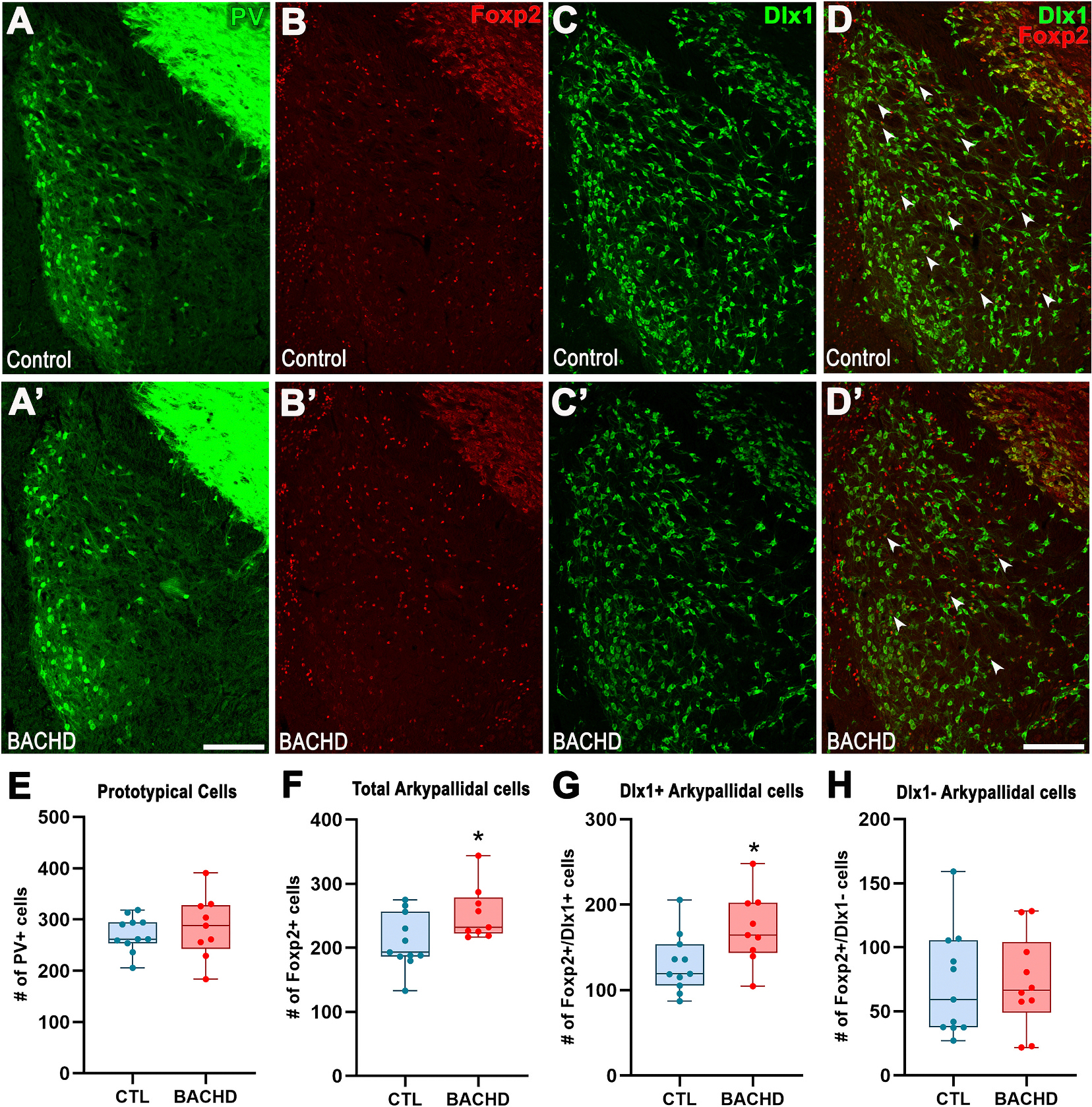
Dlx1^+^ arkypallidal GABAergic projection neurons, but not prototypical neurons, are increased in the PND13 BACHD Globus Pallidus. (A/A’–D/D′) Representative immunofluorescence images of the Globus Pallidus from PND13 mice stained for the prototypical neuron marker, PV^+^ cells (A/A’), the arkypallidal neuron marker, Foxp2 (B/B′), EGFP driven by the *Dlx1* promoter (C/C′), and combined Foxp2/Dlx1 double labeling (D/D′). (E–H) Quantification of PV^+^ prototypical (E), all Foxp2^+^ arkypallidal cells (F), Foxp2^+^/Dlx1^+^ arkypallidal cells (G), and Foxp2^+^/Dlx1^−^ cells (H). Data are presented as box-and-whisker plots showing median, interquartile range, and minimum/maximum values. Individual data points represent biological replicates. Statistical comparisons were performed using unpaired Student’s *t*-test; *p* < 0.05 was considered significant. Scale bars: 200 μm (A, D).

The globus pallidus (GP) contains two principal classes of GABAergic projection neurons: PV^+^ prototypical neurons and Foxp2-positive (Foxp2^+^) arkypallidal neurons (Dodson et al., 2015). Quantification revealed comparable numbers of PV^+^ prototypical neurons in control and BACHD mice (Control: 271.7 ± 10.3, n = 11; BACHD: 266.9 ± 29.9, n = 9; t_18_ = 0.17, *p* = 0.870; Fig. 3A/A′,E). By contrast, Foxp2^+^ arkypallidal neurons were significantly increased in BACHD mice relative to controls (Control: 209.6 ± 12.9, n = 11; BACHD: 252.9 ± 13.9, n = 9; t_18_ = 2.64, p = 0.036; Fig. 3B/B′,F).

Using the Dlx1-EGFP reporter, we identified two subpopulations of arkypallidal neurons, Dlx1-EGFP–positive and –negative (Fig. 3C/C′,D/D′). The increase in arkypallidal neurons in BACHD mice was confined to the Dlx1-EGFP–positive subpopulation (Control: 130.8 ± 10.3, n = 11; BACHD: 172.0 ± 13.9, n = 9; t_18_ = 2.43, *p* = 0.026; Fig. 3B/B′–D/D′,G), whereas Dlx1-EGFP–negative neurons showed no significant change (Control: 78.8 ± 8.9, n = 11; BACHD: 80.9 ± 7.9, n = 9; t_18_ = 0.17, *p* = 0.865; Fig. 3B/B′–D/D′,H). These findings indicate a selective expansion of a Dlx1-EGFP^+^ subpopulation of arkypallidal neurons. Although arkypallidal neurons are thought to arise predominantly from extra-MGE subpallial sources, with only a subset derived from the MGE, the selective expansion of the Dlx1-EGFP^+^ Foxp2^+^ subpopulation is consistent with aberrant ventral MGE contribution to this lineage.

Because Foxp2 is also expressed by striatal medium spiny neurons (MSNs), we examined whether the apparent increase in Foxp2^+^ neurons in the GP might reflect misclassified MSNs. We immunostained for DARPP32, a phosphoprotein highly specific to MSNs and expressed throughout their somata and processes (Supplementary Fig. S3). DARPP32 immunoreactivity in both the BACHD and control GP corresponded exclusively to MSN axonal processes traversing the nucleus; the somata of GP GABAergic projection neurons did not express DARPP32. Importantly, none of the Foxp2^+^ neurons in the GP co-labeled with DARPP32, confirming that the Foxp2^+^ population quantified here represents bona fide arkypallidal neurons rather than MSNs.

### 3.3. Selective persistence of cortical SST^+^ interneuron deficits and compensation of striatal interneuron populations in BACHD mice at PND30

**Fig. 4.**
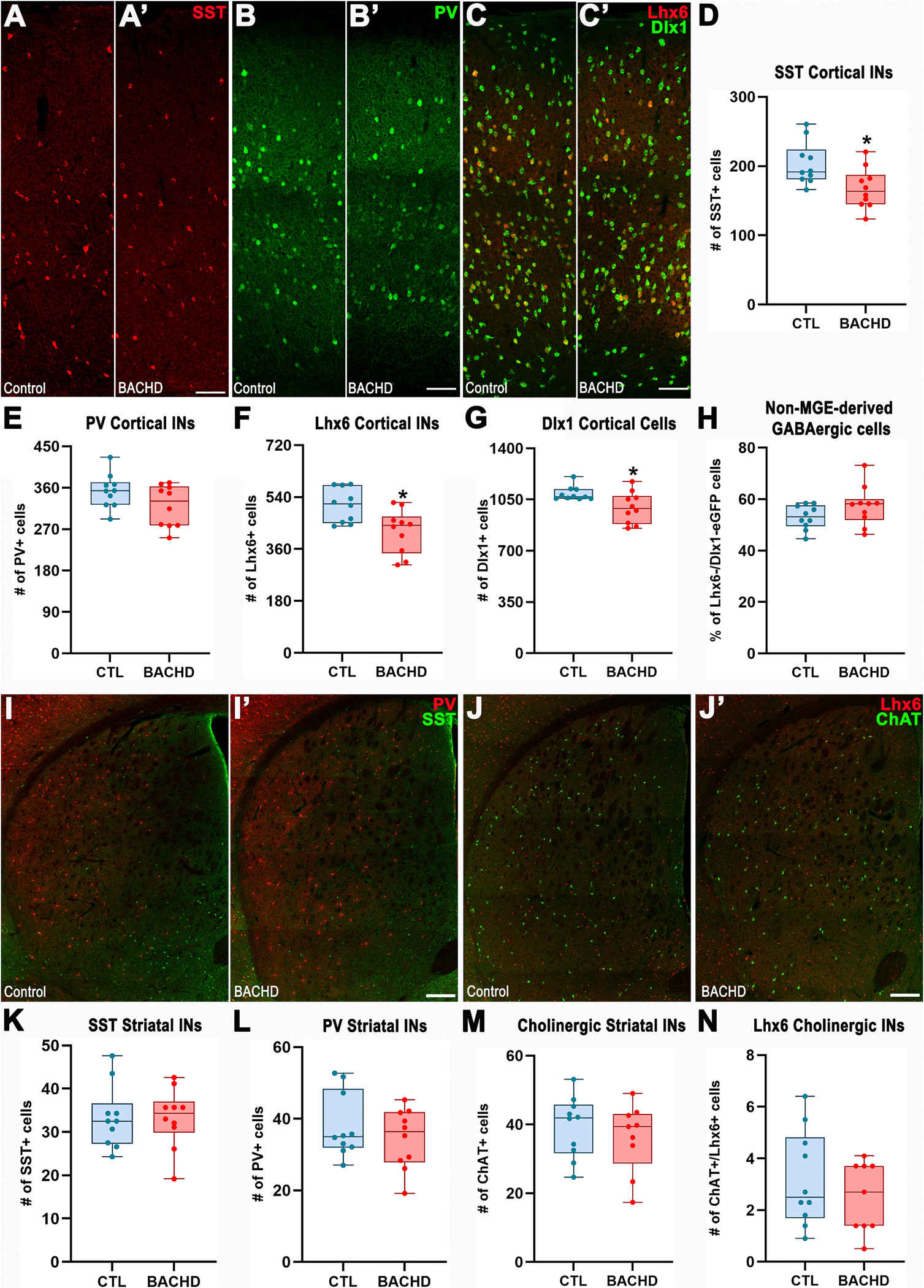
Only cortical SST interneurons are reduced in PND30 BACHD mice. Representative immunofluorescence images of the somatosensory cortex (A–C) and striatum (I–J) from PND30 mice stained for SST (A/A’), PV (B/B′), Lhx6 and Dlx1 (C/C′), PV and SST (I), and double Lhx6/ChAT (J). Cortical quantifications of SST (D), PV (E), Lhx6 (F), EGFP driven by the *Dlx1* promoter (G), and the proportion of non-MGE-derived cortical GABAergic neurons (H). Striatal quantifications of SST (K), PV (L), ChAT (M), and double Lhx6^+^/ChAT^+^ (N). Data are presented as box-and-whisker plots indicating median, interquartile range, and minimum/maximum values. Individual data points represent biological replicates. Statistical comparisons were performed using unpaired Student’s *t*-test (D-F, H, and K-N) and Mann-Whitney *U* test (G, L); *p* < 0.05 was considered significant. Scale bars: 100 μm (A–C) and 250 μm (I–J).

While the neurogenesis of forebrain interneurons is completed by late embryonic stages, their migration, integration into target cortical and striatal regions, and final maturation are not fully realized until postnatal day 30 (PND30) (Magno et al., 2021; Okaty et al., 2009; Pan et al., 2019). Accordingly, we quantified cortical and striatal interneurons at PND30. At this stage, the BACHD cortex exhibited a significant reduction in SST^+^ interneurons compared to controls (Control: 203.4 ± 9.8, *n* = 10; BACHD: 167.6 ± 9.2, *n* = 10; *t*_18_ = 2.67, *p* = 0.0157; Fig. 4A/A’,D), whereas PV^+^ interneuron numbers were not significantly different (Control: 353.1 ± 11.8, *n* = 10; BACHD: 320.4 ± 14.3, *n* = 10; *t*_18_ = 1.767, *p* = 0.094; Fig. 4B/B′,E). Unlike at PND13, the BACHD cortex at PND30 also showed a reduction in Lhx6^+^ (Control: 512.1 ± 18.6, *n* = 10; BACHD: 421.3 ± 23.8, n = 10; *t*_18_ = 2.98, *p* = 0.007; Fig. 4C/C′,F) and Dlx1^+^ cells (Control: 1090 ± 15.1, *n* = 10; BACHD: 994 ± 33.6, *n* = 10; Mann–Whitney *U* = 20, *p* = 0.023; Fig. 4C/C′,G). By contrast, the proportion of non-MGE-derived GABAergic cells remained comparable between BACHD and control cortices (Control: 53.04 ± 4.7, *n* = 10; BACHD: 57.4 ± 7.72, *n* = 10; *t*_18_ = 1.52, *p* = 0.147; Fig. 4C/C′,H). Taken together, these findings suggest that early neurogenesis deficits affecting the PV^+^ lineage are compensated by PND30, whereas deficits in the SST^+^ lineage persist, leading to a lasting reduction in SST^+^ interneuron numbers.

Conversely, neither SST^+^ (Control: 33.4 ± 2.3, *n* = 10; BACHD: 33.2 ± 2.2, *n* = 10; *t*_18_ = 0.04, *p* = 0.965; Fig. 4I/I′, K) nor PV^+^ (Control: 38.1 ± 2.9, *n* = 10; BACHD: 34.4 ± 2.6, *n* = 10; Mann–Whitney *U* = 42.5, *p* = 0.592; Fig. 4I/I′,L) interneuron numbers differed in the PND30 striatum. Similarly, no significant changes were observed in the total number of striatal cholinergic interneurons (Control: 39.3 ± 2.8, *n* = 10; BACHD: 36.1 ± 3.3, *n* = 9; *t*_17_ = 0.72, *p* = 0.479; Fig. 4J/J’,M) or in the proportion of these cells co-expressing Lhx6 (Control: 3.2 ± 0.6, *n* = 10; BACHD: 2.5 ± 0.4, *n* = 9; *t*_17_ = 0.92, *p* = 0.370; Fig. 4J/J’,N). These data indicate that earlier quantitative alterations in striatal PV^+^ and Lhx6^+^ cholinergic interneurons are compensated by PND30.

### 3.4. Reduced interneuron precursors in LGE mantle and ventral pallium with early precursor changes in the developing globus pallidus of E12.5 BACHD embryos

**Fig. 5.**
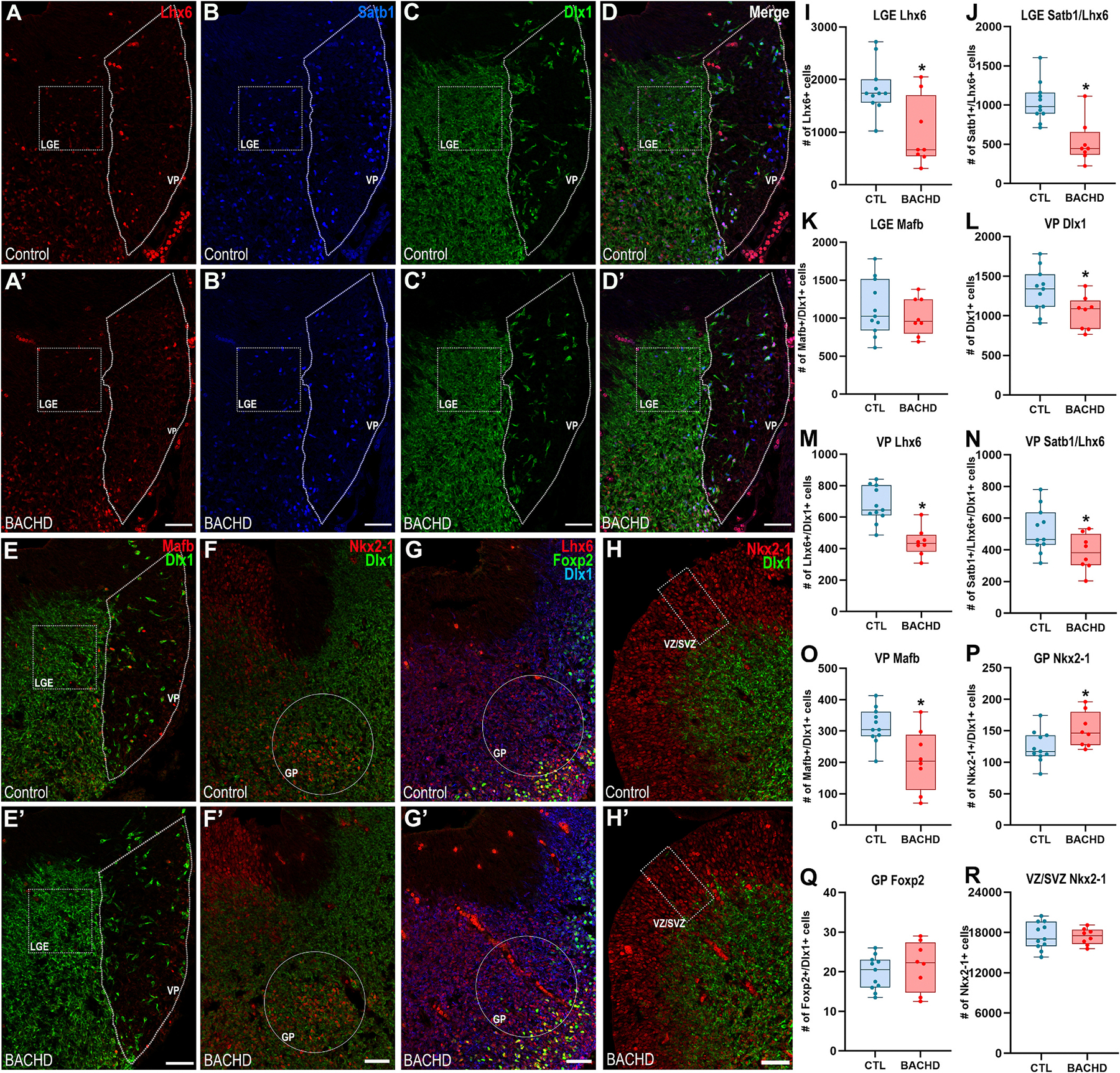
The E12.5 BACHD subpallium displays selective alterations of GABAergic precursor cells. Representative immunofluorescence images of the lateral ganglionic eminence [LGE] and ventral pallium [VP] (A–D, A’–D′), the primordium of the globus pallidus (GP; F–G, F′–G’), and the medial ganglionic eminence (MGE) germinative zone (H/H′). Tissue sections were immunostained for Lhx6 (A/A′), Satb1 (B/B′), EGFP driven by the *Dlx1* promoter (C/C′), merged staining in A to C (D/D′), Lhx6/Dlx1 (E/E′), Nkx2–1/Dlx1 (F/F′), Lhx6/Foxp2/Dlx1 (G/G′), and VZ/SVZ Nkx2–1/Dlx1 (H/H′). Quantification analyses included LGE mantle: Lhx6^+^ (I), Satb1^+^/Lhx6^+^ (J), and Mafb^+^ (K); VP: EGFP^+^ driven by the *Dlx1* promoter (L), Lhx6^+^ cells (M), Satb1^+^/Lhx6^+^ (N), and Mafb^+^ (O); Globus Pallidus (GP): Nkx2–1^+^ (P) and Foxp2^+^/Lhx6^+^ (Q) cells; and MGE VZ/SVZ Nkx2–1^+^ progenitors (R). Data are presented as box-and-whisker plots showing the median, interquartile range, and minimum/maximum values. Individual data points represent biological replicates. Statistical comparisons were performed using Mann-Whitney *U* test (I,J,Q) and unpaired Student’s *t*-test (K, L-P, R); *p* < 0.05 was considered significant. Scale bars: 50 μm.

Maturation of interneurons, including colonization into specific cortical layers, follows a time-dependent pattern correlated with birthdate (Miyoshi and Fishell, 2011). Thus, interneuron lineages that express neurochemical differentiation markers by PND13 are likely derived from early neurogenic waves. To investigate potential neurogenesis deficits affecting early-born GABAergic lineages, we examined via immunostaining techniques the E12.5 subpallium with a battery of cell lineage markers. This analysis is complicated due to the fact that the MGE subventricular and mantle zones are densely populated by progenitor and precursor cells present at diverse stages of lineage maturation with many of them sharing expression of the relevant interneuron lineage markers. To overcome this challenge, we targeted selective ventral telencephalic areas outside of the MGE, exhibiting discrete populations of MGE-derived migrating cells displaying a more homogeneous maturational stage: the LGE mantle zone, and the Ventral Pallium. The overall number of Lhx6^+^ precursor cells, were significantly reduced within the LGE mantle (Control: 1831 ± 143.1, *n* = 11; BACHD: 985.7 ± 230.9, *n* = 8; Mann–Whitney *U* = 18, *p* = 0.031; Fig. 5A/A’,D/D’,I). Reduction of Lhx6^+^ precursors appears to occur at the expense of Satb1^+^/Lhx6^+^ precursors (Control: 1037 ± 76.7, *n* = 11; BACHD: 523.5 ± 97.4, *n* = 8; Mann–Whitney *U* = 8, *p* = 0.0016; Fig. 5B/B′, D/D′,J) but not of *Mafb* (Control: 1130 ± 111.5, *n* = 11; BACHD: 1021 ± 87.8, *n* = 8; *t*_17_ = 0.723, *p* = 0.479; Fig. 5E/E’,K), the former marker enriched in SST interneuron precursors, while the latter in PV interneuron precursors.

On embryonic day 12.5 (E12.5), the ventral pallium is traversed by tangentially migrating interneurons destined for the cortex. In this region, BACHD specimens exhibited a significant reduction in Dlx1^+^ GABAergic cells compared to controls (Control: 1315 ± 83.6, *n* = 11; BACHD: 1040 ± 75.1, *n* = 8; *t*_17_ = 2.34, *p* = 0.032; Fig. 5C/C′,L). This decrease was primarily due to a loss of Lhx6^+^ MGE-derived precursor cells (Control: 677.2 ± 34.7, *n* = 11; BACHD: 441.1 ± 32.1, n = 8; *t*_17_ = 4.81, *p* = 0.0002; Fig. 5A/A’,C/C′,M), and included both Satb1^+^/Lhx6^+^ putative SST precursors (Control: 521.9 ± 42.9, *n* = 11; BACHD: 384 ± 40.6, *n* = 8; *t*_17_ = 2.25, *p* = 0.038; Fig. 5B/B′,D/D′,N) and Mafb^+^ putative PV precursors (Control: 316.2 ± 17.4, *n* = 11; BACHD: 207.9 ± 34.8, *n* = 8; *t*_17_ = 3.02, *p* = 0.007; Fig. 5E/E’,O). These findings further define neurogenesis deficits that specifically affect the generation of interneuron precursors undergoing tangential migration into the dorsal telencephalon.

We next analyzed the E12.5 caudo-external MGE mantle zone, a region encompassing the emerging globus pallidus. In this region, the number of precursor cells co-expressing *Nkx2*–*1* and *Dlx1* was significantly higher in BACHD specimens compared with controls (Control: 124 ± 7.6, n = 11; BACHD: 151.3 ± 9.9, n = 8; t_17_ = 2.23, *p* = 0.039; Fig. 5F/F′,P). By contrast, the total number of Foxp2+/Dlx1+ cells, as well as the proportion of these cells co-expressing *Lhx6*, did not differ between genotypes (Foxp2^+^/Dlx1^+^: Control: 19.7 ± 1.3, *n* = 11; BACHD: 21.4 ± 2.2, *n* = 8; *t*_17_ = 0.72, *p* = 0.473; Lhx6^+^/Foxp2^+^/Dlx1+: Control: 4.68 ± 0.54, *n* = 11; BACHD: 4.9 ± 1.1, *n* = 8; Mann–Whitney *U* = 38.5, *p* = 0.671; Fig. 5G/G’,Q). These data suggest early precursor abnormalities within the developing BACHD globus pallidus.

We then quantified ventricular/subventricular zone uncommitted progenitors expressing *Nkx2*–*1* but lacking *Dlx1* expression. The abundance of these progenitors was comparable between BACHD and control specimens (Control: 17,492 ± 610.6, *n* = 11; BACHD: 17,384 ± 431.3, *n* = 8; *t*_17_ = 0.133, *p* = 0.895; Fig. 5H/H′,R), indicating that MGE precursor cell alterations are not accompanied by changes within the progenitor populations from which these cells arise.

### 3.5. Single-cell transcriptomic analysis delineates MGE neurogenic lineages and identifies early chromatin-associated defects in BACHD progenitors

**Fig. 6.**
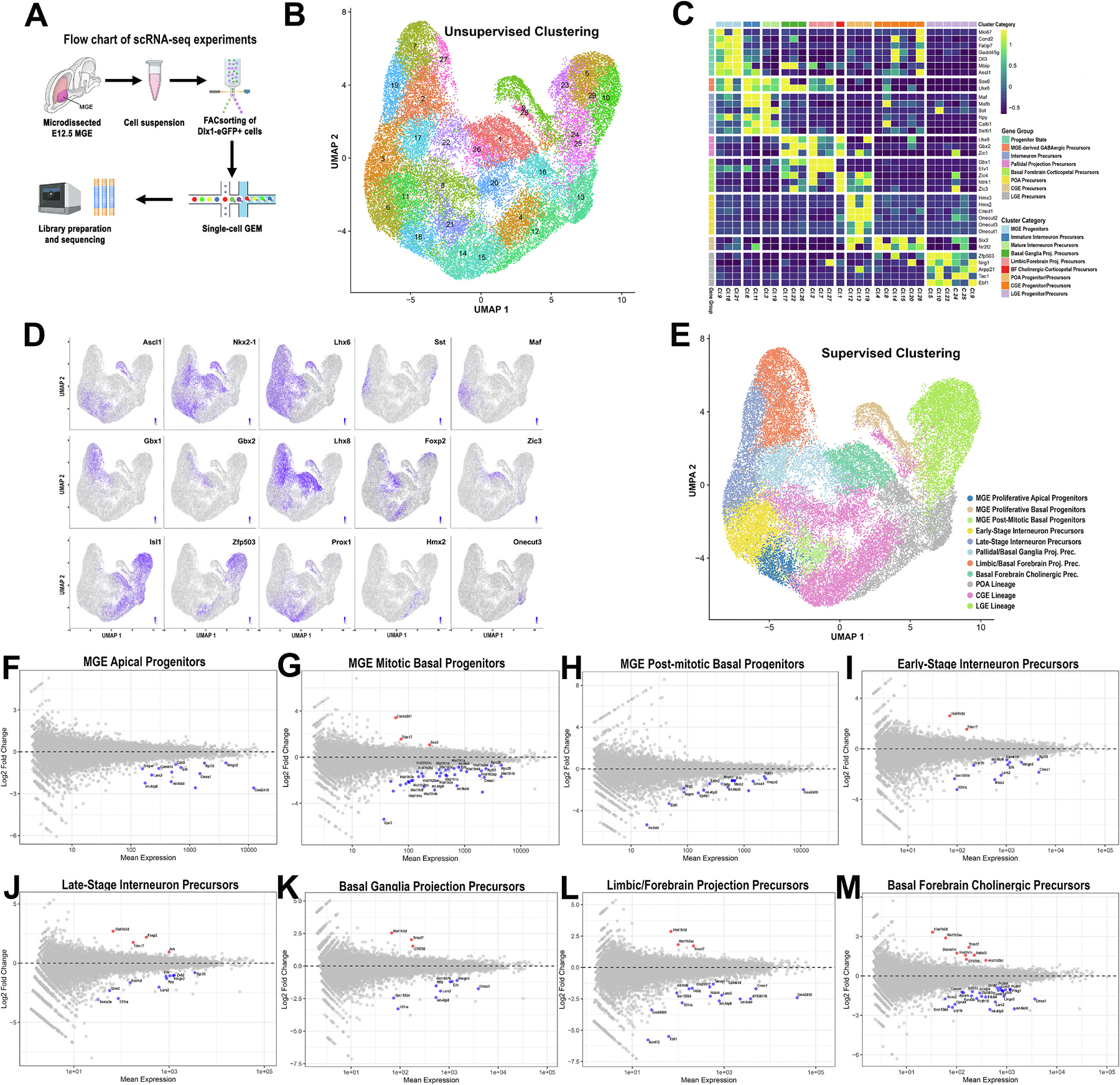
Single-cell transcriptomics reveals disrupted MGE neurogenesis and lineage specification in BACHD embryos. (A) Schematic representation of the experimental workflow for single-cell sorting based on Dlx1-EGFP expression from dissociated cell suspensions obtained by microdissection of E12.5 MGEs (two independent sets of four paired MGEs per experimental group). (B) Uniform Manifold Approximation and Projection (UMAP) of 43,504 single cells (*n* = 22,401 BACHD; *n* = 21,103 control) resolved 29 transcriptionally distinct clusters. (C) Heatmap of differentially expressed subpallial canonical marker genes (rows) across major clusters, and (D) feature plots of representative lineage-defining genes enabled supervised cluster annotation into discrete cell categories (E). (F–M) MA plots depicting differentially expressed genes between BACHD and control embryos, estimated by pseudobulk differential expression analysis using DESeq2. Genes meeting significance criteria (FDR < 0.05) are shown, with red and blue dots indicating significantly upregulated and downregulated genes, respectively. Phase-resolved chromatin and histone abnormalities identified by pseudobulk and single-cell module scoring analyses are presented in Supplementary Fig. S6. (For interpretation of the references to colour in this figure legend, the reader is referred to the web version of this article.)

We generated single-cell transcriptional profiles of GABAergic-enriched Dlx1-EGFP reporter–positive E12.5 MGE cells (Fig. 6A). After quality control, 43,504 cells (22,401 BACHD and 21,103 control cells) were retained for analysis. Unsupervised dimensionality reduction and clustering resolved 29 transcriptionally distinct populations (Fig. 6B). Based on canonical marker expression (Fig. 6C, D) these clusters were consolidated into 11 major developmental lineages through supervised classification, comprising 8 MGE-derived cell categories and 3 non-MGE lineages (Fig. 6E).

Along the primary MGE neurogenic trajectory, *Ascl1*^+^ progenitors segregated into three sequential states: proliferative apical radial glia (cl.21), enriched for *Nes* and *Fabp7*; proliferative basal intermediate progenitors (cl.9), expressing *Insm1* and *Zeb2* but lacking *Nes*; and postmitotic MGE progenitors (cl.18), characterized by induction of *Sox6* and *Lhx6* and downregulation of cell cycle–associated transcripts. Together, these transitions delineate the canonical radial glia → intermediate progenitor → postmitotic MGE lineage progression.

Beyond the post-mitotic node, the trajectory bifurcated into interneuron and projection neuron fates. Along the interneuron branch, two successive precursor stages were resolved. Early-stage interneuron precursors (cl.6, 11) exhibited a SoxD-driven transcriptional program (*Sox2*, *Sox5*, *Sox6*), together with *Satb1*, *Calb1*, *Erbb4*, *Maf/Mafb*, *Npy*, and *Lhx6*, consistent with newly specified cortical interneuron identities. A late-stage branch comprised differentiated migratory interneuron precursors (cl.3, 19), in which expression of *Sst* and *Reln*, together with synaptic and axon-associated genes (*Cntnap2*, *Dab1*, *Casz1*, *Mef2c*), marked acquisition of long-range migratory competence and early subtype diversification.

The projection neuron branch resolved into three transcriptionally discrete MGE-derived precursor classes. Basal ganglia GABAergic projection precursors (cl.17, 22, 26) expressed a coordinated *Lhx6/Lhx8, Shh, Bcl11b, Foxp1, Foxp2, Gbx2* transcriptional program, consistent with canonical globus pallidus trajectories. A second branch comprised limbic/forebrain–projecting GABAergic precursors (cl.2, 7, 27), defined by co-expression of *Lhx6/Lhx8* with *Cnr1*, *Nrp2*, *Etv1*, and *Gbx1*. A third, molecularly independent branch consisted of basal forebrain cholinergic and corticopetal precursors (cl.1), marked by a distinct cholinergic transcriptional module (*Zic1/3/4*, *Ntrk1*, *Tnr*).

Trajectory mapping also revealed migratory streams entering the MGE field from adjacent germinative zones, including preoptic area (POA) precursors (cl.12, 13, 16; *Hmx2/3*, *Cited1*, *Onecut1/2/3*, *Zfhx3/4*), caudal ganglionic eminence (CGE) precursors (cl.4, 8, 14, 15, 20, 28; *Nr2f1/2*, *Prox1*, *Sp8/9*), and lateral ganglionic eminence (LGE) precursors (cl.5, 10, 23, 24, 25, 29; *Meis2*, *Zfp503*, *Isl1*, *Ebf1*, *Nrg1*, *Arpp21*, *Tac1*). These populations likely represent tangentially migrating early-born neurons and progenitors that transiently intermingle with the MGE during E12.5 neurogenesis.

To determine how mutant HTT disrupts early MGE neurogenesis, we performed pseudobulk differential expression analyses across all major MGE cell categories in E12.5 BACHD and control embryos (Fig. 6F–M). Across the progenitor continuum, including apical radial glia, basal intermediate progenitors, and early progenitor cells exiting the cell cycle, we identified a shared transcriptional signature marked by downregulation of mitochondrial respiratory chain genes (*mt-Nd4l, mt-Atp8, mt-Nd5*), mitochondrial translation factors (*Cmss1, Lars2*), and ribosomal components (*Rpl35, Rps28, Rps29*), indicating early bioenergetic and biosynthetic insufficiency within the MGE progenitor pool (Fig. 6F–H).

Basal intermediate progenitors exhibited the most pronounced chromatin-associated defects, characterized by broad repression of canonical replication-dependent histone genes spanning the HIST1 and HIST2 clusters (*Hist1h1a*–*d; Hist1h2ab/ae/ai/ap/bb/bj/bm; Hist1h3b/c/e/f; Hist1h4c/d/h; Hist2h2ac*), together with reduced expression of *Erh*, a chromatin-associated regulator of cell cycle progression and genome stability (Fig. 6G). Gene Ontology enrichment analyses supported this chromatin-centered phenotype, revealing overrepresentation of Biological Process terms related to nucleosome positioning and assembly, chromatin assembly and remodeling, DNA packaging, chromosome organization, and histone H3K27 and H3K4 methylation, as well as Cellular Component terms associated with nucleosome, chromatin, chromosome, and euchromatin organization (Supplementary Fig. S4). These defects are accompanied by sustained *Sox3* expression and reduced expression of signaling and adhesion molecules associated with neurogenic competence (*Gpc5, Cdh18, Tac1, Egfem1, Sema6d, Il31ra*), together with increased expression of the stress-linked E3 ubiquitin ligase *Trim17*.

Consistent with the scRNA-seq findings, immunohistochemical analyses revealed a significant increase in Sox3^+^ progenitors in BACHD embryos within both the ventricular zone (Control: 14,874 ± 828, *n* = 5; BACHD: 18,710 ± 703, *n* = 4; Mann–Whitney *U* = 0, *p* = 0.015; Supplementary Fig. S5E) and subventricular zone (Control: 15,060 ± 1091, *n* = 5; BACHD: 19,304 ± 918, *n* = 4; Mann–Whitney *U* = 0, *p* = 0.015; Supplementary Fig. S5F), but not within the intermediate zone (Control: 10,328 ± 2143, *n* = 5; BACHD: 12,968 ± 2698, *n* = 4; Mann–Whitney *U* = 5, *p* = 0.285; Supplementary Fig. S5G). BrdU incorporation assays demonstrated a selective increase in S-phase–labeled progenitors within the subventricular zone of BACHD embryos (Control: 12,031 ± 1176, *n* = 5; BACHD: 14,988 ± 974, *n* = 4; Mann–Whitney *U* = 0, *p* = 0.016; Supplementary Fig. S5I), whereas BrdU^+^ cell numbers were comparable between genotypes in the ventricular zone (Control: 6783 ± 2100, *n* = 5; BACHD: 7995 ± 1734, *n* = 4; Mann–Whitney *U* = 4, *p* = 0.191; Supplementary Fig. S5H) and intermediate zone (Control: 5242 ± 955, *n* = 5; BACHD: 5133 ± 1052, *n* = 4; Mann–Whitney *U* = 9, *p* = 0.904; Supplementary Fig. S5J). In contrast, pHH3^+^ mitotic indices did not differ between control and BACHD embryos across any MGE germinative zone (ventricular zone: Control: 1028 ± 510, *n* = 5; BACHD: 1027 ± 708, *n* = 4; Mann–Whitney *U* = 9, *p* = 0.904; subventricular zone: Control: 568 ± 274, *n* = 5; BACHD: 407 ± 173, *n* = 4; Mann–Whitney *U* = 7, *p* = 0.556; intermediate zone: Control: 484 ± 105, *n* = 5; BACHD: 382 ± 200, *n* = 4; Mann–Whitney *U* = 8, *p* = 0.73; Supplementary Fig. S5K–M).

To distinguish whether chromatin-associated transcriptional defects reflected altered cell cycle composition or phase-intrinsic dysregulation, basal intermediate progenitors were analyzed using continuous transcriptional scores corresponding to G1-like, S, and G2/M phases. G1-like cells were operationally defined as actively cycling progenitors in early G1, characterized by low S-phase and G2/M-phase transcriptional scores together with robust enrichment of proliferation-associated genes, thereby excluding quiescent (G0) states. Violin plot analysis revealed increased dispersion of S-phase scores and a pronounced rightward skew of G2/M scores specifically in BACHD basal, but not apical, progenitors, indicating a broader distribution of cells with elevated replication- and late cell cycle–associated transcriptional signatures (Supplementary Fig. S6A,B). These altered score distributions closely parallel the selective increase in BrdU^+^ S-phase progenitors observed in vivo, despite unchanged mitotic indices. Consistent with these findings, phase-specific cell counts revealed a marked depletion of BACHD basal progenitors in the G1-like compartment (15.3% [95% CI, 13.01–18.04] vs 29.09% [95% CI, 25.9–29.1] in controls; Fisher’s exact test, *p* < 0.0001), accompanied by a reciprocal enrichment in G2/M-phase cells (60.8% [95% CI, 57.3–64.1] vs 46.8% [95% CI, 43.2–46.8]; Fisher’s exact test, *p* < 0.0001) (Supplementary Fig. S6C). Together, these data indicate selective disruption of cell cycle progression in BACHD basal progenitors, with accumulation at late cell-cycle stages and reduced representation of the G1-like compartment.

Phase-restricted pseudobulk analyses demonstrated that repression of canonical replication-dependent histone genes is already evident in S-phase BACHD basal progenitors and not only persists but intensifies in G2/M, as reflected by increased statistical strength and broader representation across histone subclasses (Supplementary Fig. S6E,F; Supplementary Data 4). Consistent with these phase-resolved transcriptional effects, single-cell module scoring revealed reduced expression of a downregulated histone module, defined empirically by histone genes identified as repressed in BACHD basal progenitors by pseudobulk analysis, across both S and G2/M phases (Supplementary Fig. S6G). To further assess whether histone repression extended beyond replication-coupled nucleosomal histones, a parallel analysis restricted to Histone-1 family members was performed, as linker histones regulate higher-order chromatin organization and are less strictly replication-coupled. Histone-1 family members exhibited a highly similar pattern, with reduced Histone-1 module scores in BACHD basal progenitors during both S and G2/M phases (Supplementary Fig. S6H). Together, these findings indicate that histone repression in BACHD basal progenitors reflects a phase-intrinsic chromatin regulatory abnormality rather than a secondary consequence of altered cell-cycle dynamics.

In contrast, G1-like cells in both genotypes exhibited minimal histone module expression, consistent with the replication-coupled regulation of these genes. Notably, G1-like BACHD basal progenitors displayed robust upregulation of genes not normally expressed in E12.5 Dlx1^+^ MGE progenitors, including *Foxp2*, *Tbr1*, and *Slc17a6*, together with early differentiation-associated factors *Lhx2*, *Lhx1/Lhx1os*, and *Samd3* (Supplementary Fig. S6D; Supplementary Data 4). As scRNA-seq was performed on FACS-sorted Dlx1-EGFP^+^ cells, these changes reflect aberrant transcriptional programs within MGE-derived progenitors rather than contamination by non-MGE or post-mitotic neuronal populations.

Newly post-mitotic progenitors were further distinguished by broad suppression of neural developmental transcription factors and guidance molecules (*Ebf1*, *Nrg3*, *Ephb1*, *Nxph1*, *Hs3st4*, *Tnik*, *Nfib*, *Meis2*) and the spindle/centrosome regulator, *Aspm* (Fig. 6H). Conversely, across interneuron and basal projection precursor lineages, BACHD embryos exhibited convergent transcriptional changes characterized by continued downregulation of mitochondrial (*mt-Atp8*, *mt-Nd4l*, *Lars2*) and chromatin-associated (e.g., *Hist1h3d*) genes, reduced *Erh*, and increased *Trim17* expression (Fig. 6I-M).

Among late-stage interneuron precursor subtypes, *Zeb2*, a key regulator of the cortical–striatal fate switch (McKinsey et al., 2013), was significantly downregulated, suggesting impaired specification of cortical interneuron identity (Fig. 6J). Conversely, *Foxp2*, more typically associated with GABAergic projection neuron lineages (Schreiweis et al., 2019), was increased, consistent with partial engagement of alternative lineage-associated transcriptional features. In addition, *Nfib* and *Ebf1*, transcriptional regulators implicated in neurogenic maturation, were downregulated in basal ganglia–projecting and limbic/forebrain–projecting GABAergic precursor populations, respectively (Fig. 6K, L). Finally, basal-cholinergic precursors exhibited coordinated downregulation of genes linked to synaptic signaling (*Erbb4*, *Grid2*, *Cpne4*, *Car10*), neuronal polarity (*Pard3b*), adhesion and wiring specificity (*Pcdh7/9/10*, *Alcam*, *Cdh13*), and excitability maturation (*Kcnc2*, *Kcnip4*), a pattern consistent with impaired progression toward a differentiated, connectivity-competent neuronal state (Fig. 6M).

In summary, single-cell analysis of the E12.5 MGE resolves a structured neurogenic hierarchy and identifies basal intermediate progenitors as the population showing the most pronounced transcriptional changes in BACHD embryos. These changes include coordinated alterations in mitochondrial, ribosomal, and replication-associated chromatin gene expression, together with altered S-phase labeling. Related transcriptional alterations are also observed in post-mitotic interneuron and GABAergic projection neuron precursors, affecting genes associated with differentiation and subtype specification across multiple MGE-derived lineages.

### 3.6. Genetic rescue of MGE-derived interneurons prevents motor deficits in BACHD mice

**Fig. 7.**
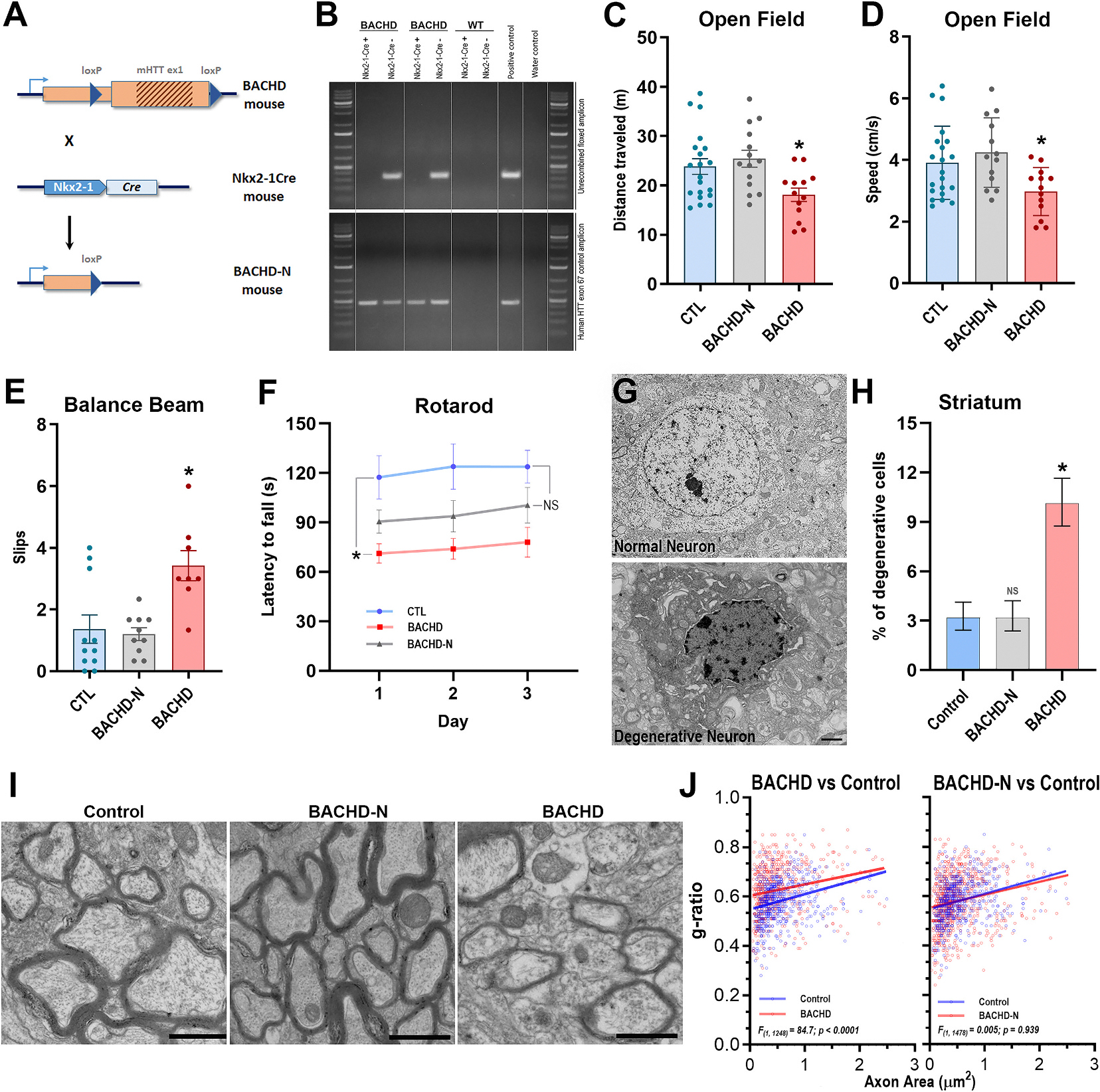
Genetic rescue of the Nkx2–1 lineage ameliorates motor deficits, striatal pathology, and myelin deficits in BACHD mice. (A) Schematic representation of Cre-mediated excisional recombination of mutant huntingtin (mHTT) in the Nkx2–1 lineage of BACHD mice. (B) PCR analyses confirming excision of human *HTT* exon 1 within the Nkx2–1 lineage of BACHD-N mice. Dissociated forebrain cells from BACHD;Nkx2–1-Cre;tdTomato and WT;Nkx2–1-Cre;tdTomato mice were FACS-sorted, and genomic DNA from tdTomato^+^ and tdTomato^−^ fractions was analyzed for the unrecombined floxed sequence (top; recombination assay) and for human *HTT* exon 67 (bottom; genotype control). Non–FACS-sorted genomic DNA from a BACHD mouse served as a positive control. The positive control corresponds to non-FACS-sorted DNA from a BACHD specimen. (C-F) Motor performance of 12-month-old mice assessed using the Open Field (C, D), Balance Beam (E), and Accelerating Rotarod (F) test paradigms. (G) Representative images of striatal neurons exhibiting normal or degenerative morphologies used for quantification. (H) Quantification of normal and degenerative neurons per biological replicate (*n* = 5, 5, and 4 for CTL, BACHD, and BACHD-N, respectively; all 12 months of age). Neurons were counted across 50 open square areas (6400 μm^2^ each) of the copper grids and pooled to build a contingency table. (I) Representative micrographs of myelinated axons within the deep region of somatosensory cortical layer 6. (J) Scatter plots comparing myelin g-ratios among control, BACHD, and BACHD-N mice (axon data from each experimental group was pooled; *n* = 5, 6, and 9, respectively). Statistical comparisons in (C-E) were performed with one-way ANOVA, (F) repeated measures ANOVA, (H) Fisher’s exact test, (J) Extra sum-of-squares *F* test; *p* < 0.05 was considered significant. Bars in (C-E) represent mean ± standard deviation, and (H) represent percentages ±95% confidence intervals (CI). Latency to fall at each day interval (F) represents mean ± standard deviation. Scale bars: 1 μm.

Cumulative evidence indicates that interneuron deficits during critical periods of forebrain circuit formation lead to long-lasting alterations, including impaired thalamocortical feedforward inhibition onto pyramidal cells, cortico-striatal hyperactivity and hyperconnectivity, and marked changes in striatal synaptic plasticity (Kozorovitskiy et al., 2012; Peixoto et al., 2019; Peixoto et al., 2016). Perturbations of this nature are likely to play an important role in HD pathogenesis. To directly assess the contribution of MGE–derived interneurons to motor dysfunction, a core feature of HD, we selectively eliminated mutant huntingtin (mHTT) expression from this lineage in BACHD mice on a C57BL/6 J background via Cre-mediated excisional recombination of the mutant allele (Fig. 7A). BACHD mice, which carry the full-length human mHTT transgene with a floxed exon 1, were crossed with mice expressing Cre recombinase under the control of the Nkx2–1 promoter, a defining marker of the MGE patterning domain. The resulting genetically rescued mice, hereafter referred to as BACHD-N, express mHTT in all forebrain cell populations except those derived from the MGE, in which the mutant allele is excised, effectively generating a null mHTT state. Excisional recombination was validated in FACS–sorted forebrain cells from BACHD-N mice carrying the Rosa26^LSL-tdTomato reporter by PCR amplification of the sequence encompassing loxP-flanked region (Fig. 7B). In contrast to tdTomato-negative cells, no discernible unrecombined floxed amplicon was detected in Nkx2–1–lineage cells, demonstrating efficient Nkx2–1-Cre–driven recombination.

Backcrossing onto C57BL/6 J substantially reduced the excessive weight gain characteristic of BACHD FVB/N mice (Supplementary Fig. S7). At 12 months, male WT, BACHD-N, and BACHD mice weighed 29.9, 31.4, and 32.9 g, respectively (*n* = 16, 13, 11; Kruskal–Wallis = 4.84, *p* = 0.088), and female WT, BACHD-N, and BACHD mice weighed 26.4, 28.6, and 33.6 g (*n* = 18, 15, 8; Kruskal–Wallis = 11.4, *p* = 0.033; WT vs. BACHD-N *p* = 0.99; WT vs. BACHD *p* = 0.022). Although BACHD females remained heavier than WT, their weight was well below the ~45 g typical of BACHD FVB/N females and within the normal C57BL/6 J range (Cummings et al., 2009). Importantly, genetic rescue of Nkx2–1 lineages normalized the weight gain in BACHD-N mice. Because Nkx2–1 is also expressed in hypothalamic progenitors, this rescue may have prevented previously reported hypothalamic circuit alterations (Dickson et al., 2022; Hult et al., 2011; Soylu-Kucharz et al., 2016).

Motor function was assessed in 12-month-old mice using the Open Field (Fig. 7C,D), Balance Beam (Fig. 7E), and Accelerating Rotarod (Fig. 7F) tests. In the Open Field test, total distance traveled differed significantly among BACHD, BACHD-N, and control groups (18.1 ± 1.4, 25.4 ± 1.7, and 23.8 ± 1.6, *n* = 13, 14, and 20, respectively; one-way ANOVA, *F*_(2,44)_ = 5.03, *p* = 0.011). BACHD mice traveled shorter distances than controls (Tukey’s HSD, *p* = 0.038, 95% CI = 0.26 to 11.3), whereas BACHD-N mice did not differ from controls (*p* = 0.756, 95% CI = −6.97 to 3.79). Ambulation speed also differed significantly across groups (BACHD, BACHD-N and control 2.97 ± 0.21, 4.23 ± 0.31, and 3.91 ± 0.26, [*n* = 13, 13, 21] respectively; one-way ANOVA, *F*_(2,44)_ = 4.93, *p* = 0.011). Only BACHD mice exhibited significantly reduced speeds compared to controls (Tukey’s HSD, *p* = 0.047, 95% CI = 0.009 to 1.85), whereas BACHD-N mice performed comparably (*p* = 0.655, 95% CI = −1.25 to 0.58). These findings indicate that genetic rescue of the MGE lineage prevents the emergence of the hypokinetic phenotype in BACHD mice.

Balance Beam performance also differed significantly among groups (mean slips: 4.56 ± 1.2, 1.45 ± 0.32, and 1.36 ± 0.46 for BACHD, BACHD-N, and controls [*n* = 9, 11, 11], respectively; one-way ANOVA, *F*_(2,28)_ = 6.29, *p* = 0.006; Fig. 7E). Compared to controls, BACHD mice displayed more slips (Tukey’s HSD, *p* = 0.01, 95% CI = −5.69 to −0.69), whereas BACHD-N mice did not (*p* = 0.99, 95% CI = −2.45 to 2.27). Rotarod performance (*n* = 8, 12, and 12 for BACHD, BACHD-N, and controls, respectively; Fig. 7F) revealed a significant group effect on latency to falls (*F*(_2,29)_ = 5.34, *p* = 0.011). Post hoc analysis showed that BACHD mice had shorter latencies than controls (Dunnett’s test, *p* = 0.0064, 95% CI = 12.86 to 81.83), whereas BACHD-N mice performed similarly to controls (*p* = 0.094, 95% CI = −4.04 to 57.65). No differences were observed by day (*F*(_2,58)_ = 2.28, *p* = 0.110) or group*day (*F*_(2,58)_ = 182, *p* = 0.946). Together, these results demonstrate that selective removal of mHTT from the MGE lineage forestalls the development of motor coordination deficits in BACHD mice.

### 3.7. Genetic rescue of the MGE lineage prevents striatal degeneration, corrects myelination deficits, and prevents early interneuron deficits in BACHD mice

Striatal degeneration is another hallmark of HD. We therefore examined whether genetic rescue of the MGE lineage could mitigate this pathology. Striatal specimens from 12-month-old BACHD, BACHD-N, and control mice (*n* = 5, 4, and 5, respectively; Fig. 7G,H) were processed for transmission electron microscopy. Neurons were counted in 50 open square areas (6400 μm^2^ each) per specimen and classified as having normal or degenerative morphologies. Counts from each specimen were pooled to construct a contingency table. The proportion of degenerative striatal neurons was significantly higher in BACHD mice compared to controls (10.1% [95% CI, 8.8–11.7] vs. 3.2% [95% CI, 2.4–4.1]; Fisher’s exact test, *p* < 0.0001; Fig. 7H). In contrast, BACHD-N mice had a percentage of degenerative neurons comparable to controls (3.2% [95% CI, 2.4–4.2] vs. 3.2% [95% CI, 2.4–4.1]; Fisher’s exact test, *p* > 0.999). These results indicate that MGE-lineage genetic rescue prevents striatal degeneration in BACHD mice.

We next assessed the impact of MGE-lineage rescue on axonal hypomyelination, another consistent pathological feature of HD. Myelin g-ratios were quantified in the deep region of somatosensory cortical layer 6 in 12-month-old control, BACHD, and BACHD-N mice (*n* = 5, 6, and 9, respectively; Fig. 7I,J). For linear regression analysis, axons were pooled by group (*n* = 631, 620, and 850 for control, BACHD, and BACHD-N, respectively). BACHD mice exhibited a clear hypomyelination phenotype compared to controls (intercept: *F*_(1,1248)_ = 84.7, *p* < 0.0001; slope: *F*_(1,1247)_ = 1.67, *p* = 0.196). Remarkably, myelination in BACHD-N mice was indistinguishable from controls (intercept: *F*_(1,1478)_ = 0.005, *p* = 0.939; slope: *F*_(1,1477)_ = 0.579, *p* = 0.447), suggesting that non–cell-autonomous mechanisms involving MGE-derived cells contribute to myelin deficits in BACHD.

Given the PV^+^ and SST^+^ interneuron deficits observed at PND13 in BACHD mice, we next asked whether early genetic rescue of mutant huntingtin (mHTT) in Nkx2–1–expressing MGE progenitors prevents the emergence of these abnormalities. Quantitative analyses revealed that control and BACHD-N mice exhibited comparable numbers of PV^+^ and SST^+^ interneurons in both the cortex (PV^+^: Control: 195.2 ± 57.9, *n* = 10; BACHD-N: 221.1 ± 68.8, *n* = 10; *t*_18_ = 0.91, *p* = 0.375; Supplementary Fig. S8A/A‘,E; SST^+^: Control: 256.1 ± 50.0, *n* = 11; BACHD-N: 275.6 ± 34.4, *n* = 10; *t*_19_ = 1.03, *p* = 0.314; Supplementary Fig. S8B/B′, F) and the striatum (PV^+^: Control: 18.2 ± 3.7, *n* = 11; BACHD-N: 20.1 ± 3.8, *n* = 11; *t*_20_ = 1.19, *p* = 0.245; Supplementary Fig. S8C/C′,G; SST^+^: Control: 50.1 ± 6.1, *n* = 11; BACHD-N: 49.7 ± 6.2, *n* = 11; *t*_20_ = 0.13, *p* = 0.896; Supplementary Fig. S8D/D′,H). These findings indicate that removal of mHTT from the MGE lineage is sufficient to prevent early PV^+^ and SST^+^ interneuron deficits, supporting a lineage-autonomous requirement for mHTT expression in driving these developmental abnormalities and reinforcing their relevance to Huntington’s disease pathogenesis.

## 4. Discussion

Using the BACHD model, we show that disrupted developmental neurogenesis of MGE-derived GABAergic lineages represents an early pathogenic vulnerability and that perturbation of these lineages plays a necessary role in later emergence of key HD hallmarks. At E12.5, the subpallium exhibits selective vulnerabilities across interneuron lineages, with early reductions biased toward SST-associated precursors and more spatially restricted effects on PV-lineage progenitors within migratory corridors, alongside a reciprocal increase in globus pallidus GABAergic projection neuron precursors. These developmental imbalances are evident during the first two postnatal weeks but partially resolve by PND30, with the notable exception of a persistent deficit in cortical SST^+^ interneurons, paralleling previously reported early cortico-striatal circuit disturbances (Cepeda et al., 2025b). Occurring during a critical period of circuit formation, these early disruptions are positioned to exert lasting effects on network function, including cortical hyperexcitability, a recognized feature of HD (Cepeda et al., 2025a; Nardone et al., 2007; Orth et al., 2010; Schippling et al., 2009). Consistent with a causal role, targeted genetic rescue of MGE lineages prevented both motor decline and striatal neurodegeneration.

Beyond their role in maintaining excitatory–inhibitory balance in mature circuits, interneurons play essential roles in developmental plasticity and circuit refinement (Le Magueresse and Monyer, 2013; Reh et al., 2020; van Versendaal and Levelt, 2016). Early-born SST interneurons act as pioneer cells that scaffold thalamocortical inputs onto infragranular PV interneurons (Tuncdemir et al., 2016). Their loss disrupts PV maturation, weakens thalamic drive, and reduces feedforward inhibition, leading to cortico-striatal dysfunction (Kozorovitskiy et al., 2012; Peixoto et al., 2016). Multiple HD models show comparable delays in GABAergic maturation and early thalamocortical and cortico-striatal perturbations (Cepeda et al., 2025a; Cepeda et al., 2025b; Cepeda et al., 2003; Cepeda et al., 2019; Holley et al., 2022; Parievsky et al., 2017; Serranilla and Woodin, 2021). Although many of these abnormalities are transient, they are likely to engage developmental programming mechanisms with lasting consequences (Chen and Zhang, 2011; Ratie and Humbert, 2024; Vaiserman et al., 2018), and failure of later homeostatic compensation may precipitate HD phenotypes (Cepeda et al., 2025a; Cepeda et al., 2025b). Moreover, the lifelong deficit of cortical SST^+^ interneurons observed here may further potentiate hyperexcitability arising from early circuit maldevelopment, rendering cortico-striatal networks increasingly vulnerable to excitotoxic stress. It also remains possible that subtle misspecification of MGE-derived neurons contributes to enhanced susceptibility to age-dependent cell loss.

Our findings align with postmortem studies reporting interneuron deficits in HD (Kim et al., 2014; Mehrabi et al., 2016; Reiner et al., 2013), in which such abnormalities correlate with symptom heterogeneity, severity, and neuronal loss. Similar deficits and associated inhibitory circuit abnormalities, have been documented across several HD models (Cummings et al., 2009; Holley et al., 2019; Rallapalle et al., 2021; Voelkl et al., 2022; Wang et al., 2024), suggesting that interneuron dysfunction represents a conserved component of HD pathophysiology. Here, we extend these findings by demonstrating that interneuron deficits arise early in development and are evident during the first postnatal weeks. By PND13, BACHD mice already display reduced numbers of cortical SST+ and PV+ interneurons, as well as fewer striatal PV+ interneurons, despite preservation of the overall Lhx6^+^ interneuron pool. Notably, BACHD mice display quantifiable behavioral abnormalities between PND10 and PND21, including impaired ultrasonic vocalizations, hyperkinetic phenotypes, and altered risk-taking behavior (Siebzehnrubl et al., 2018) temporally coinciding with these cellular abnormalities.

Interneuron neurogenesis follows an extended timeline, producing an initial surplus of cells that are later refined by apoptosis (Denaxa et al., 2018a; Denaxa et al., 2018b; Mancia Leon et al., 2020; Miyoshi et al., 2007; Southwell et al., 2012; Wong et al., 2018). Because interneuron maturation is asynchronous and tightly linked to birthdate and lineage trajectory, selective disruption of early-born SST^+^ and PV^+^ interneurons provides a parsimonious explanation for their reduced numbers at PND13, whereas preservation of Lhx6^+^ interneurons likely reflects later-born populations that have not yet acquired mature subtype markers. The transient reduction of PV^+^ interneurons at PND13 is consistent with early loss of Mafb^+^ PV precursors at E12.5, followed by later compensatory neurogenesis, in line with the extended temporal window of PV lineage production. By PND30, PV^+^ interneuron numbers no longer differ significantly between genotypes, whereas SST^+^ interneurons remain persistently reduced, consistent with the more restricted developmental window of SST^+^ interneurons relative to PV^+^ cells, whose neurogenesis extends into late gestation (Miyoshi et al., 2007). In addition to reduced neurogenic output, delayed maturation or impaired acquisition of molecular identity may contribute to these patterns, as suggested by reports of molecular marker loss in SST^+^ and VIP^+^ interneurons in the R6/2 HD model (Voelkl et al., 2022).

We also observed elevated numbers of Nkx2–1^+^ precursors in the globus pallidus at E12.5 and increased Foxp2^+^ arkypallidal neurons at PND13. While Foxp2+ arkypallidal cells are generally thought to arise from extra-MGE sources (Dodson et al., 2015), our previous work indicated a MGE contribution of up to 10% (Mehler et al., 2019). Consistent with this, scRNA-seq clusters enriched for MGE markers (*Nkx2*–*1, Lhx6/8, Gbx1/2*) also expressed *Foxp2*, suggesting that a previously underappreciated subset of arkypallidal neurons derives from the MGE. Thus, the increased number of arkypallidal neurons may reflect aberrant expansion of a ventral MGE-derived Foxp2^+^ subpopulation. Notably, selective inactivation of *Htt* within the MGE lineage previously produced similar increases in arkypallidal neurons together with interneuron deficits (Mehler et al., 2019), implicating *Htt* loss-of-function–related mechanisms in MGE microdomain patterning and lineage specification. Such an early increase in arkypallidal neuron number may influence pallido–striatal inhibitory feedback during critical periods of basal ganglia circuit tuning during plasticity, with possible long-term consequences for action selection and motor control.

Single-cell transcriptomic analyses identify MGE basal progenitors as a core site of vulnerability to mutant HTT and link early progenitor dysfunction to downstream neuronal abnormalities. Across the MGE neurogenic continuum, mutant HTT disrupts replication-coupled chromatin regulation and biosynthetic capacity, with basal intermediate progenitors most strongly affected. Broad repression of replication-dependent histone genes, reduced *Erh* expression, and associated mitochondrial deficits indicate an impaired ability to meet the chromatin assembly demands of proliferative amplification, accompanied by sustained expression of the stress-associated factor Trim17. Consistent with this framework, cell-cycle–resolved histone module analyses further demonstrate that histone repression persists across late cell-cycle phases and extends to linker Histone-1 family members, indicating disruption of higher-order chromatin regulation rather than a simple consequence of altered S-phase dynamics. Cell cycle–resolved analyses demonstrate that these chromatin abnormalities are already evident within individual cell cycle phases and are associated with a redistribution of basal progenitors toward late cell cycle states, consistent with unresolved replication-associated chromatin stress prior to mitotic entry. In vivo cell-cycle labeling corroborates this framework, revealing selective accumulation of S-phase progenitors without increased mitotic output, indicative of impaired cell-cycle progression rather than enhanced proliferation. Together, these findings extend and mechanistically refine our earlier evidence of mutant HTT–induced progenitor cell cycle dysregulation during subpallial development (Molero et al., 2009). By resolving these abnormalities at single-cell resolution, our data position defective replication-coupled chromatin regulation upstream of altered progenitor dynamics and downstream lineage perturbations. Importantly, cell cycle stratification further reveals destabilization of progenitor identity during the G1-like stage, marked by inappropriate activation of differentiation- and region-associated transcriptional programs normally excluded from Dlx1^+^ MGE progenitors. Collectively, these findings support a model in which mutant HTT disrupts replication-coupled chromatin regulation, delays orderly cell cycle progression, and uncouples early G1 transit from lineage control, thereby propagating fate instability into post-mitotic precursors.

Our prior studies demonstrated that selective expression of mutant Htt during development is sufficient to produce motor deficits, NMDA receptor–mediated excitotoxic vulnerability, and striatal degeneration comparable to lifelong mutant Htt expression (Molero et al., 2016). Developmentally restricted partial loss of *Htt* similarly produced motor deficits, striatal cell loss, and HD-like hypomyelination (Arteaga-Bracho et al., 2016; Hickman et al., 2021). Conditional ablation of *Htt* within subpallial lineages using *Gsx2*- and *Nkx2*–*1*-Cre drivers resulted in aggressive basal ganglia degeneration and motor impairments (Mehler et al., 2019). By one month of age, these mice showed SST^+^ interneuron deficits, and by 12 months, progressive loss of striatal and cortical PV^+^ interneurons, paralleling findings in human HD (Kim et al., 2014; Reiner et al., 2013). Collectively, these studies not only emphasized the contribution of developmental alterations to HD pathogenesis but also the mechanistic involvement of MGE neurogenesis deficits. The present study extends these observations by demonstrating that genetic rescue of the Nkx2–1 MGE lineage prevents the emergence of major motor and degenerative phenotypes in BACHD mice, despite continued mutant Htt expression in other forebrain lineages, strongly implicating aberrant MGE neurogenesis in HD pathogenesis.

Finally, developmental disruptions in HD likely extend beyond neurons to oligodendrocyte lineages. Migrating interneurons regulate gliogenesis and oligodendrocyte maturation through paracrine signaling and direct interactions (Orduz et al., 2015; Voronova, 2017), and oligodendrocyte maturation and myelination are impaired in HD and *Htt* loss-of-function models (Arteaga-Bracho et al., 2016; Jin et al., 2015; Lim et al., 2022). Here, genetic rescue of MGE lineages also prevented white matter hypomyelination in BACHD mice, implicating interneuron dysfunction as a contributor to myelin pathology. Because cortical myelination during critical period closure stabilizes emerging neural circuits (Xin et al., 2024), early myelin deficits may further exacerbate circuit maldevelopment. Consistent with this view, targeting mutant Htt in oligodendrocytes via NG2-Cre improved behavioral phenotypes in BACHD mice (Ferrari Bardile et al., 2019).

In summary, our findings demonstrate that developmental impairments differentially disrupt neurogenesis of MGE-derived GABAergic lineages in BACHD mice, affecting both globus pallidus projection neurons and interneurons in the cortex and striatum. These abnormalities arise during a period of maximal circuit plasticity, and their prevention through targeted genetic rescue forestalls the emergence of core HD phenotypes, including motor impairments, striatal degeneration, and myelination deficits. Together, these data support a developmental model of HD pathogenesis in which non–cell-autonomous mechanisms driven by aberrant MGE lineage specification and maturation exert lasting effects on circuit stability and disease vulnerability, with important translational implications for early, lineage-targeted disease-modifying interventions.

## Supplementary Material

MMC1

MMC5

MMC4

MMC2

MMC9

MMC3

MMC8

MMC7

MMC6

MMC10

MMC11

MMC12

Supplementary data to this article can be found online at https://doi.org/10.1016/j.nbd.2026.107297.

## Data Availability

Data will be made available on request. Gene expression data have been deposited in the Gene Expression Omnibus (GEO) under accession number GSE316394
