# Supplementary figures and images for: Aberrant medial ganglionic eminence (MGE) GABAergic neurogenesis contributes to Huntington’s disease pathogenesis
Source: Neurobiol Dis. Author manuscript; Available in PMC 2026 Apr 23. (PMC13103998; doi:10.1016/j.nbd.2026.107297)

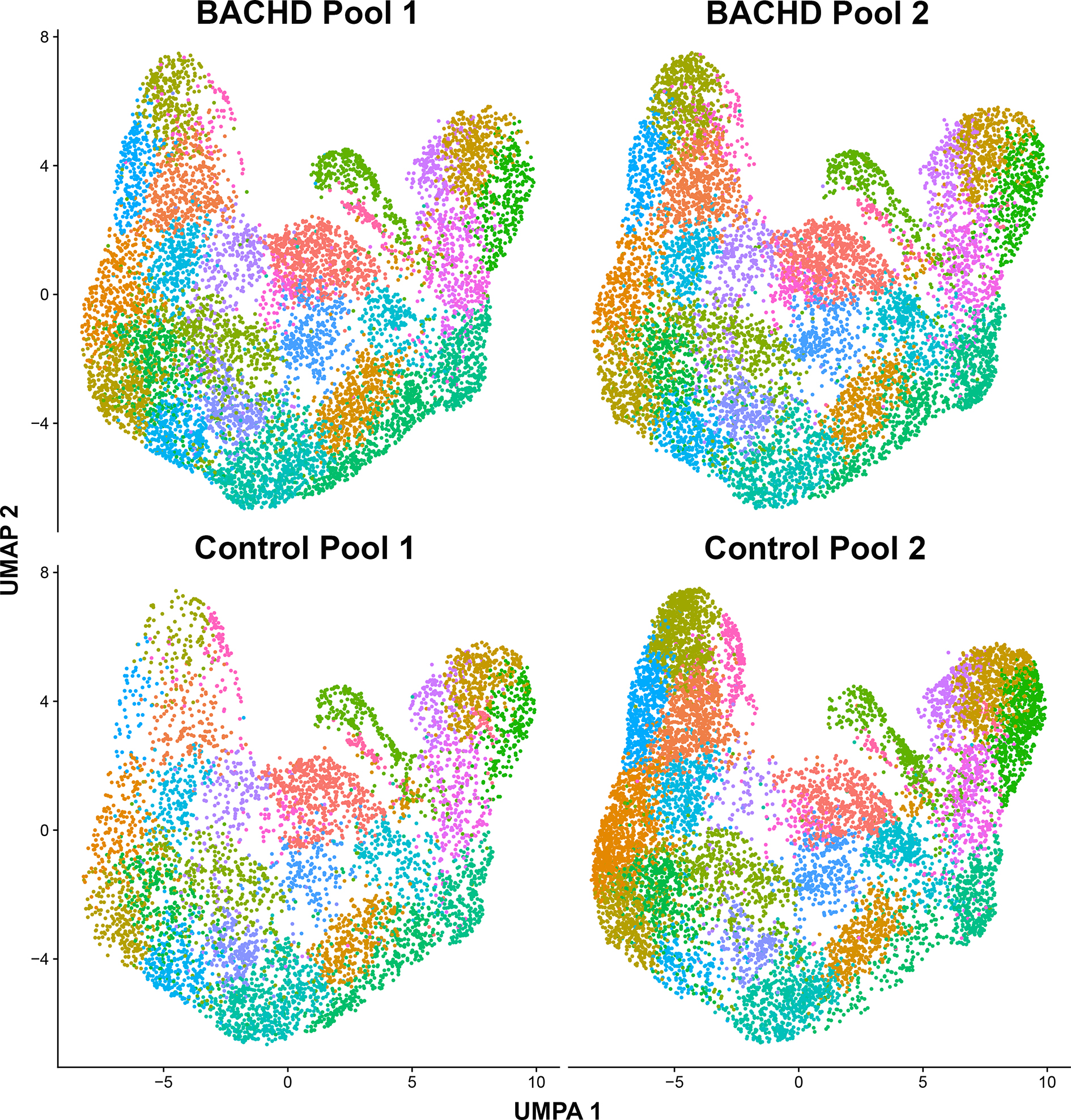

Supplement: MMC1 [file NIHMS2161138-supplement-MMC1.jpg]

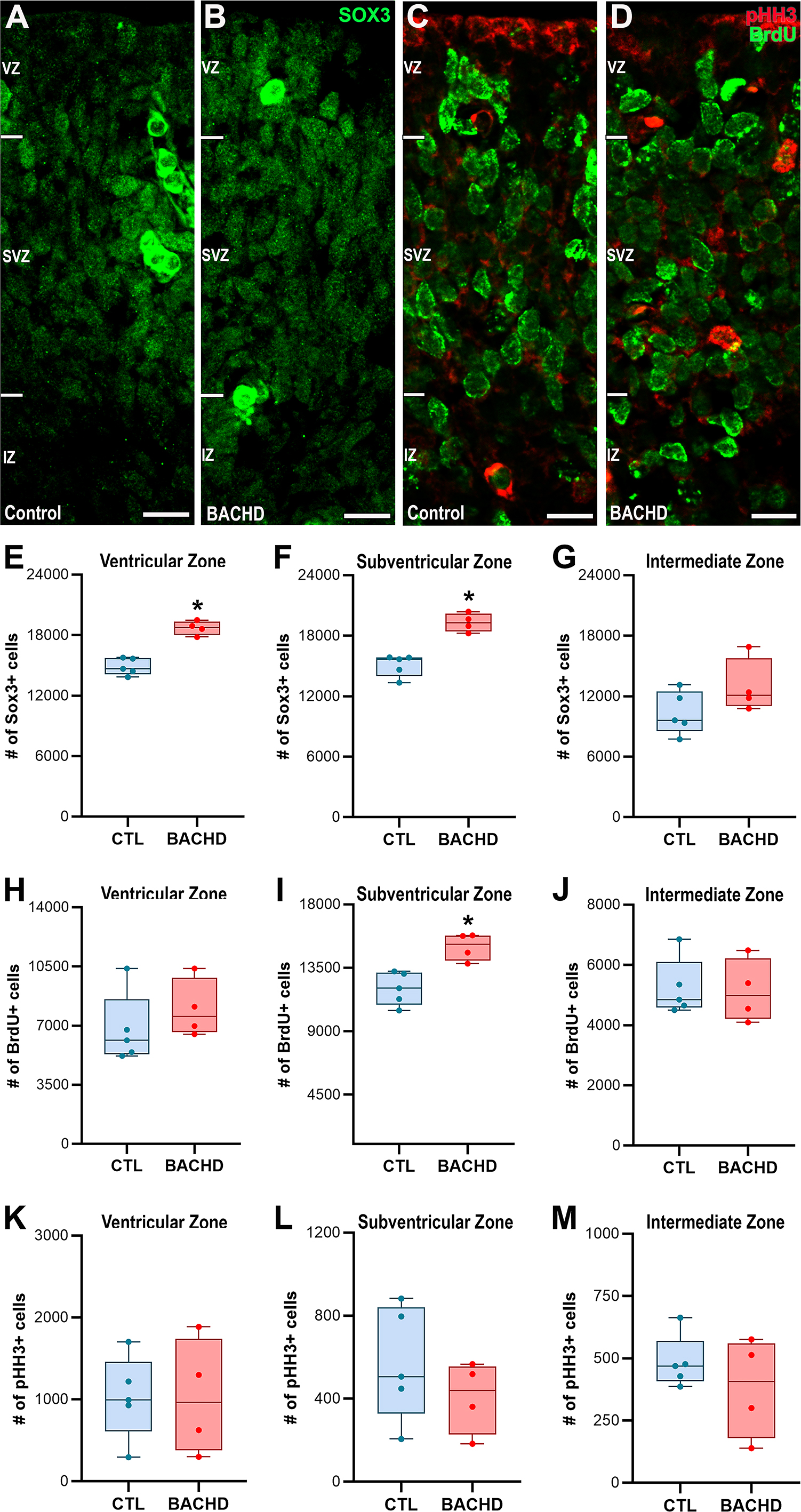

Supplement: MMC5 [file NIHMS2161138-supplement-MMC5.jpg]

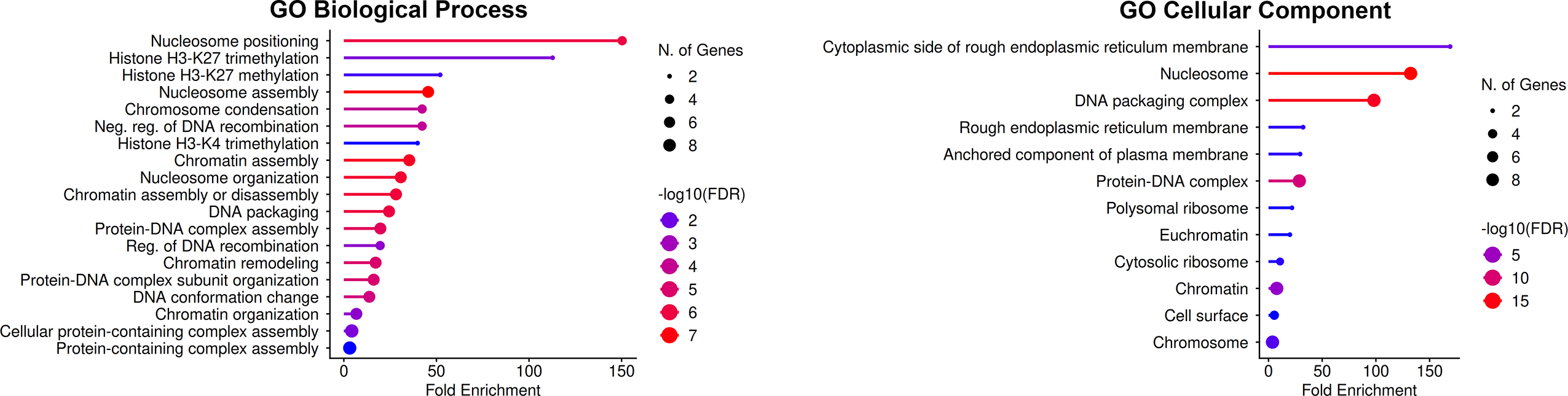

Supplement: MMC4 [file NIHMS2161138-supplement-MMC4.jpg]

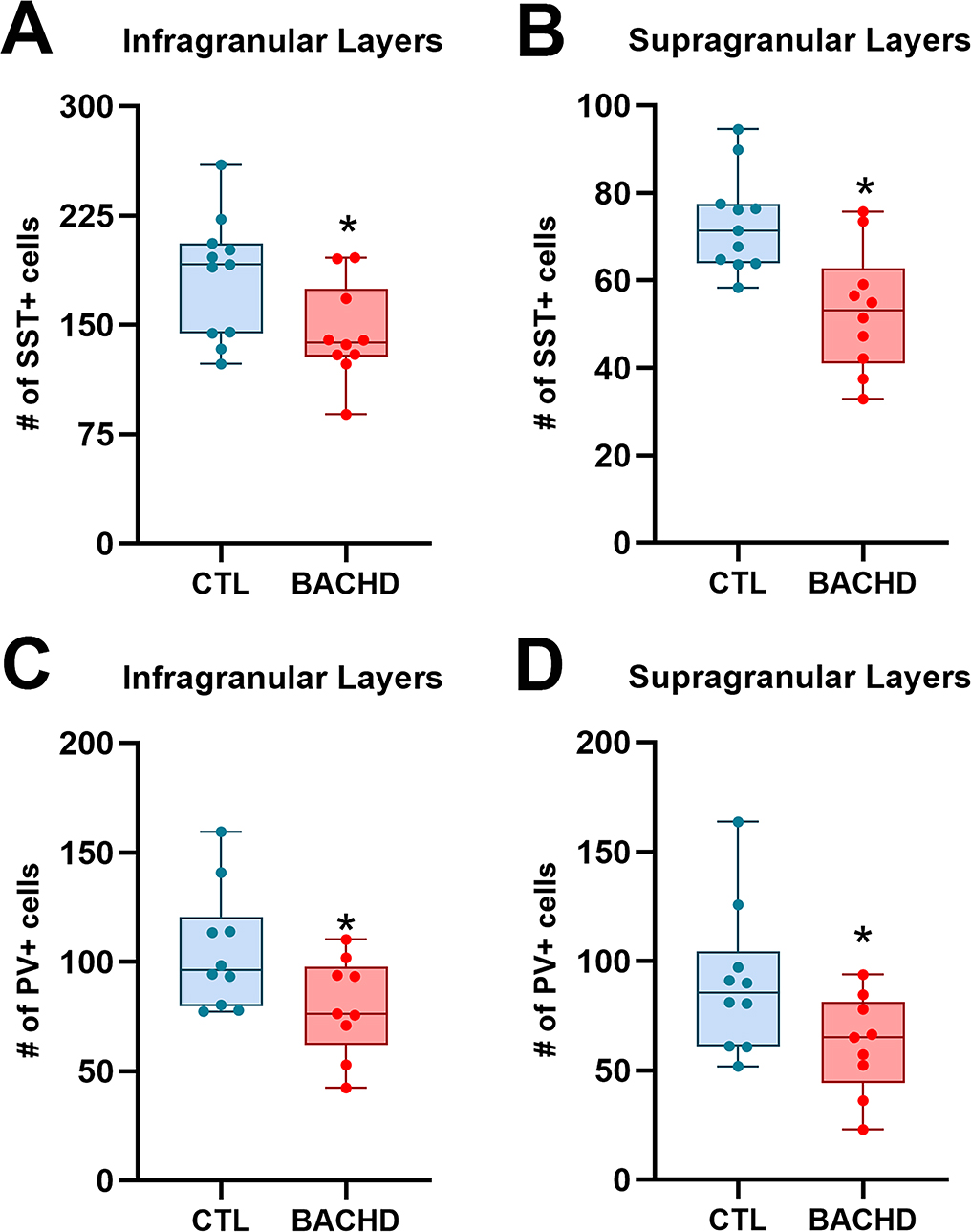

Supplement: MMC2 [file NIHMS2161138-supplement-MMC2.jpg]

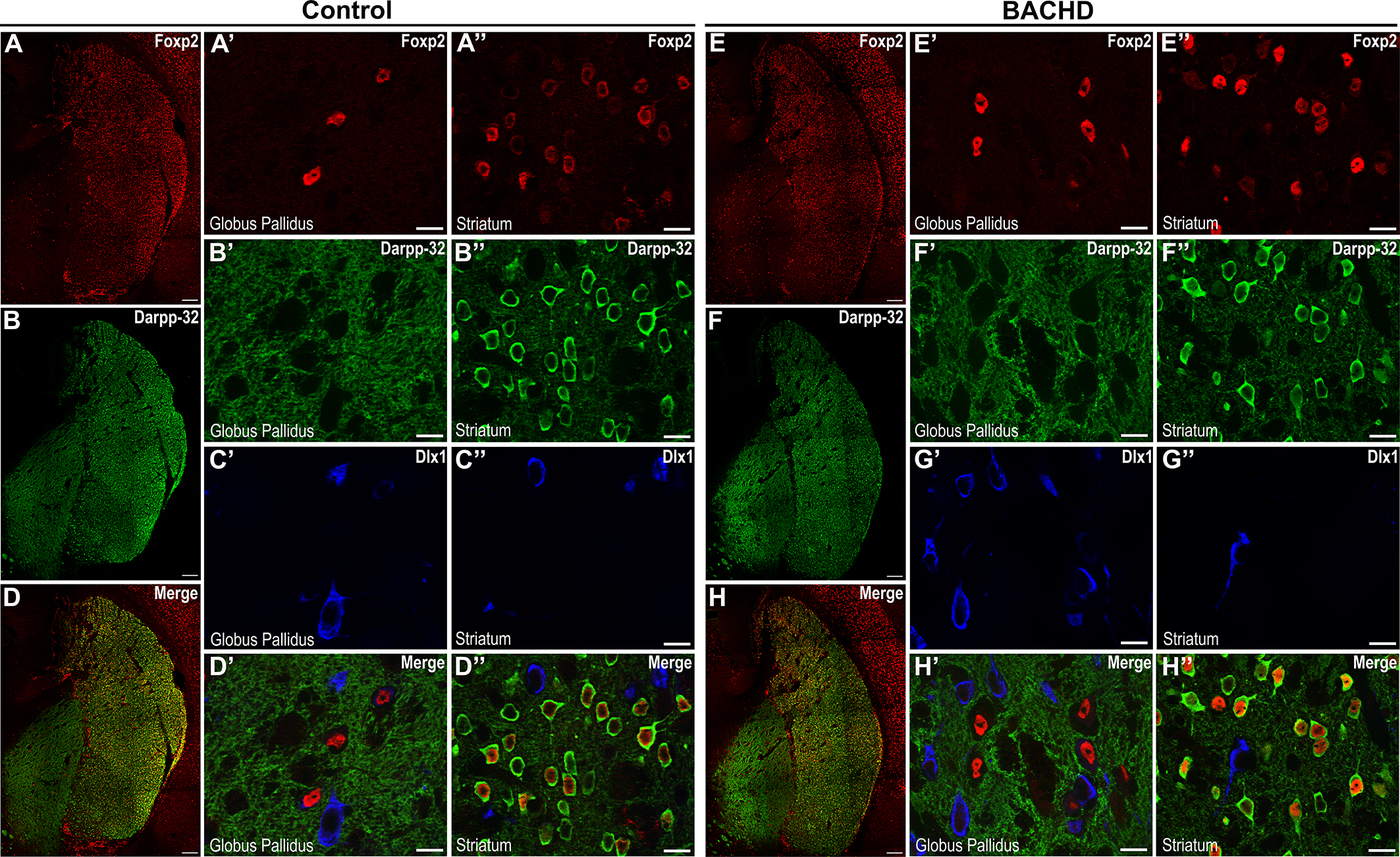

Supplement: MMC3 [file NIHMS2161138-supplement-MMC3.jpg]

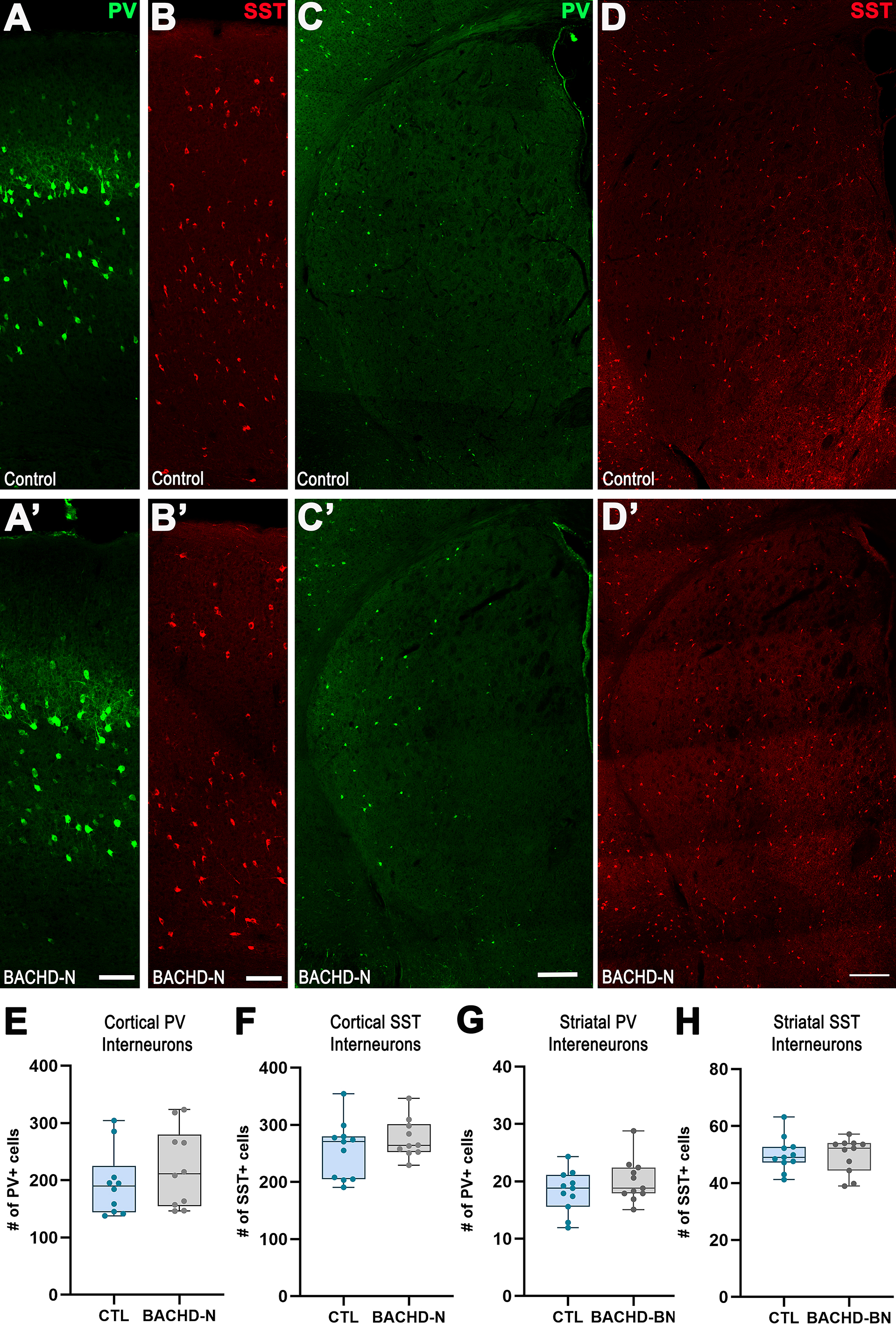

Supplement: MMC8 [file NIHMS2161138-supplement-MMC8.jpg]

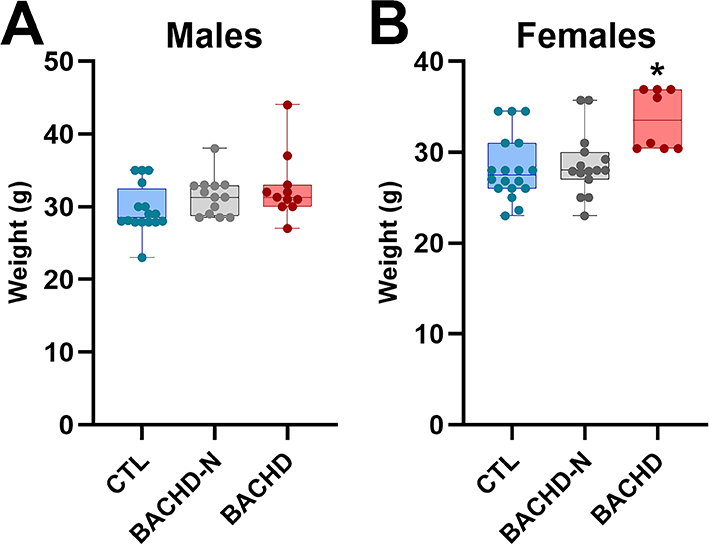

Supplement: MMC7 [file NIHMS2161138-supplement-MMC7.jpg]

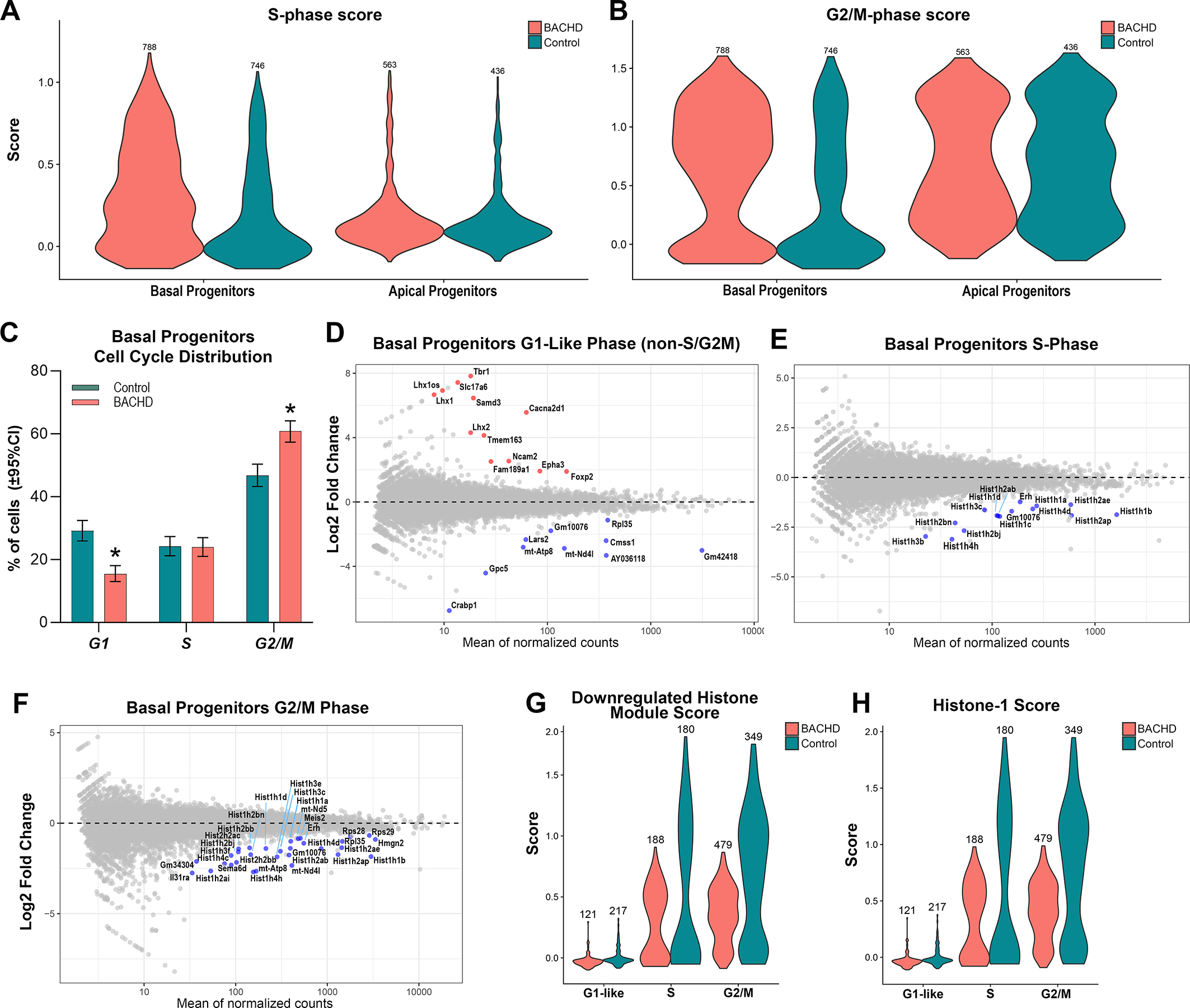

Supplement: MMC6 [file NIHMS2161138-supplement-MMC6.jpg]
